# DanHong injection targets endothelin receptor type B and angiotensin II receptor type 1 in protection against cardiac hypertrophy

**DOI:** 10.18632/oncotarget.21900

**Published:** 2017-10-13

**Authors:** Min-Yu Zhang, Fei-Fei Guo, Hong-Wei Wu, Yang-Yang Yu, Jun-Ying Wei, Shi-Feng Wang, Yu-Xin Zhang, Ming-Hua Xian, Qing-Hua Wu, Bu-Chang Zhao, Shi-You Li, Hong-Jun Yang

**Affiliations:** ^1^ Institute of Chinese Materia Medica, China Academy of Chinese Medical Sciences, Beijing, China; ^2^ Beijing Key Lab of TCM Collateral Disease Theory Research, School of Traditional Chinese Medicine, Capital Medical University, Beijing, China; ^3^ Key Laboratory of Genomic and Precision Medicine, Beijing Institute of Genomics, Chinese Academy of Sciences, Beijing, China; ^4^ School of Chinese Materia Medica, Beijing University of Chinese Medicine, Beijing, China; ^5^ Buchang Pharmacy Group, Xi'an, China

**Keywords:** cardiac hypertrophy, human induced pluripotent stem cell-derived cardiomyocytes, endothlin B receptor, angiotensin II receptor 1, DanHong injection

## Abstract

Cardiac hypertrophy (CH) is an independent risk factor for cardiovascular diseases (CVDs). Mitigating or preventing CH is the most effective strategy for the treatment of CVDs. DanHong injection (DH) is a Chinese herbal medicine preparation (CHMP) widely used in clinical treatment of several CVDs in China. However, the direct targets and cellular mechanisms for these protective effects remain unclear. This study was designed to illustrate the direct targets of DH in protecting against CH and investigate CH molecular pathogenesis. A hypertrophic cell model was induced by endothelin-1 (ET-1) on human induced pluripotent stem cell-derived cardiomyocytes (hiPS-CMs). Real time cellular analysis (RTCA) cardio system and high content analysis (HCA) were used to detect the changes in contractile function, morphology and protein level of hypertrophic hiPS-CMs. Agonist and antagonist assay on receptors were performed using calcium mobilization high-throughput screening (HTS). DH significantly attenuated CH by modulating myocardial contractility, suppressing cell area enlargement and down-regulating ET-1-induced brain natriuretic peptide (BNP), actinin alpha 2 (ACTN2) and cardiac muscle troponin T (TNNT2) protein expression (*P* < 0.05). Endothelin receptor type B (ETBR) and angiotensin II receptor type 1 (AT1R) were DH direct targets, with IC50 value of 25.67 μL/mL and 1.10 μL/mL, respectively. Proteomics analysis showed that proteins involved in cell cycle inhibition, RNA processing, mitochondrial translation and cytoskeleton are significant regulated by DH treatment. These data revealed that ETBR and AT1R are DH direct targets on protecting against CH, providing a strategy to explore direct targets of CHMPs.

## INTRODUCTION

Cardiac hypertrophy (CH) is an independent risk factor for the increased incidence and mortality of various malignant cardiovascular diseases (CVDs), such as ischemic heart disease, arrhythmia and sudden cardiac death [1]. Pathological CH is an adaptive response to long-term heart overload and to a variety of excessive stimulation, resulting in an increased cardiomyocytes volume and protein content, as well as interstitial cell proliferation [2]. Although compensatory CH can increase cardiac contractility, it will eventually develop into ventricular dilatation and heart failure (HF). On the one hand, CH is a common pathological change of various CVDs, although the pathological mechanism is still not clear yet. On the other hand, although angiotensin converting enzyme inhibitors and angiotensin receptor blockers have been used for the treatment of CH in clinical trials over the decades, more and more studies showed that the treatment above had no significant effect in many patients [3]. Hence, since no efficient treatment for CH, it is urgent to find effective target drugs.

DanHong injection (DH) is a Chinese herbal medicine preparation (CHMP), which has become a hotspot in recent years for its wide application and good curative effect on cardiovascular and cerebrovascular diseases. More than 4,000 papers focused on DH in China are available, of which 2805 are clinical studies (CNKI database and NCBI PubMed). Previous studies have showed the clinical protective effects of DH on angina pectoris (955 articles), ischemic cardiomyopathy (165 articles), and cerebral infarction (740 articles). Recently DH is increasingly used to treat chronic HF in clinical trials [4]. However, these studies rarely revealed DH protective mechanism against these malignant diseases. In modern research, the vast majority of studies have shown that DH can improve the symptoms of cardiovascular and cerebrovascular diseases mentioned above. However, no studies are available to clarify DH direct targets.

DH composition have already been analysed in previous studies. A total of 28 catechols were detected, including the major compounds of tanshinol, protocatechuic aldehyde, salvianolic acid B, rosmarinic acid, salvianolic acids A and D, and lithospermic acid after intravenous DH administration in human subjects, rats, and dogs [5]. A pharmacokinetic study suggested that coexisting Honghua constituents might have negligible influences on the pharmacokinetics of Danshen polyphenols from DH [6]. Furthermore, seven DH major components were identified as human serum albumin ligands [7]. Its major components were reported in a previous study [8]. In our study, the ingredients of DH were identified as a quality control (Supplementary Figure 1 and Supplementary Table 1). It is worth mentioning that DH was considered as a whole in the present research for its clinical efficacy.

In this study, the therapeutic effect of DH on CH was evaluated using human induced pluripotent stem cell-derived cardiomyocytes (hiPS-CMs) stimulated with endothelin-1(ET-1) to obtain CH model *in vitro*. Real time cellular analysis (RTCA) cardio system was applied to monitor hiPS-CMs function. RT-PCR and high content analysis (HCA) were used to detect the changes in gene expression, morphology and protein level of hypertrophic hiPS-CMs. A cell-based calcium mobilization high-throughput screening (HTS) assay was performed to evaluate the agonistic and antagonistic effect of DH on the receptors. Protein changes in the hiPS-CMs were analysed by proteomics techniques and bioinformatics methods.

## RESULTS

### DH attenuated ET-1 induced CH on hiPS-CMs

**Figure 1:**
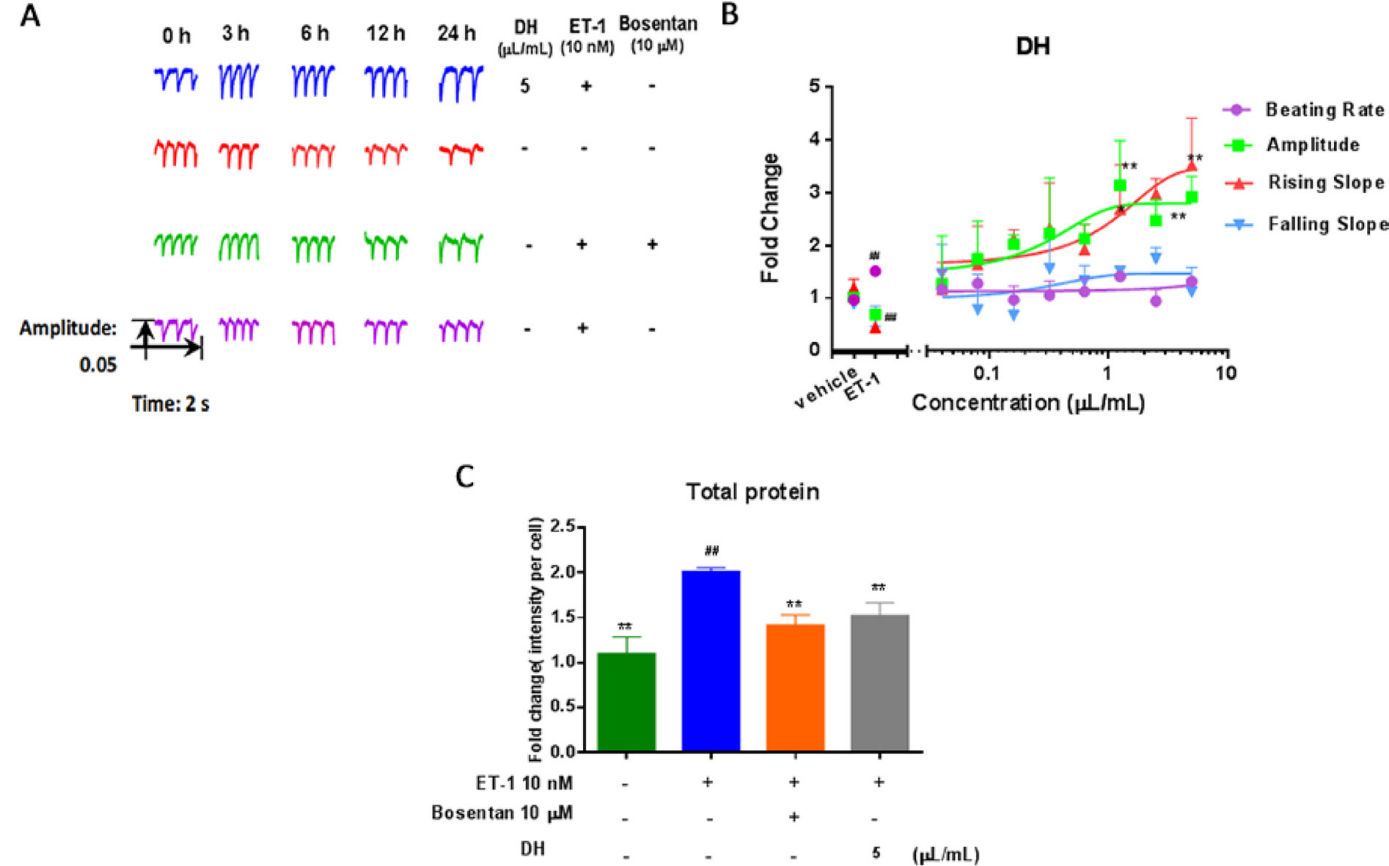
DH modulate beating patterns of hiPS-CMs in CH (**A**) Representative beating traces captured at selected time points of hiPS-CMs treated by DH. (**B**) Representative contraction profiles treated by various dose (0.04, 0.08, 0.16, 0.32, 0.63, 1.25, 2.5 and 5.0 μL/mL) of DH. Four CM beating parameters, including beating rate, amplitude, rising slope, and falling slope were used for evaluation 3.0 hours after DH treatment. (**C**) DH reduced the quantity of total protein on hypertrophic hiPS-CMs. ^**^P < 0.05: versus ET-1 group; ^##^*P* < 0.05: versus vehicle group.

DH effect on hypertrophic hiPS-CMs was evaluated using the dynamic myocardial function assay. Five parameters, including the whole beating patterns, beating rate, amplitude, rising slope and falling slope, were used to estimate hiPS-CMs contractility. Rising slope and falling slope reflected the systolic and diastolic function of the CMs, respectively [9]. Cardiac function is mainly achieved by the rhythmic contraction of cardiomyocytes. CH appeared in HF, developing negative inotropic effects manifested by a myocardial decrease in amplitude. The amplitude of hiPS-CMs was increased by DH treatment in a dose-dependent manner compared with ET-1 group. Rising slope showed an identical trend of amplitude. The beating parameters of hiPS-CMs at 3.0 h were used to evaluate the remarkable changes in contractility during real time detection (Figure 1A). As the beating traces showed, the amplitude and rising slope of hiPS-CMs were heightened by DH treatment, and no clear change in beating rate and falling slope appeared compared with ET-1 group. In the present study, DH showed time- and dose-dependent positive inotropic effects on cardiomyocytes (Figure 1B). The amplitude of hypertrophic cardiomyocytes increased in response to treatment with DH, indicating its potential cardiotonic effects. Furthermore, DH (5 μL/mL) suppressed the increase of total protein on hypertrophic hiPS-CMs (Figure 1C).

**Figure 2:**
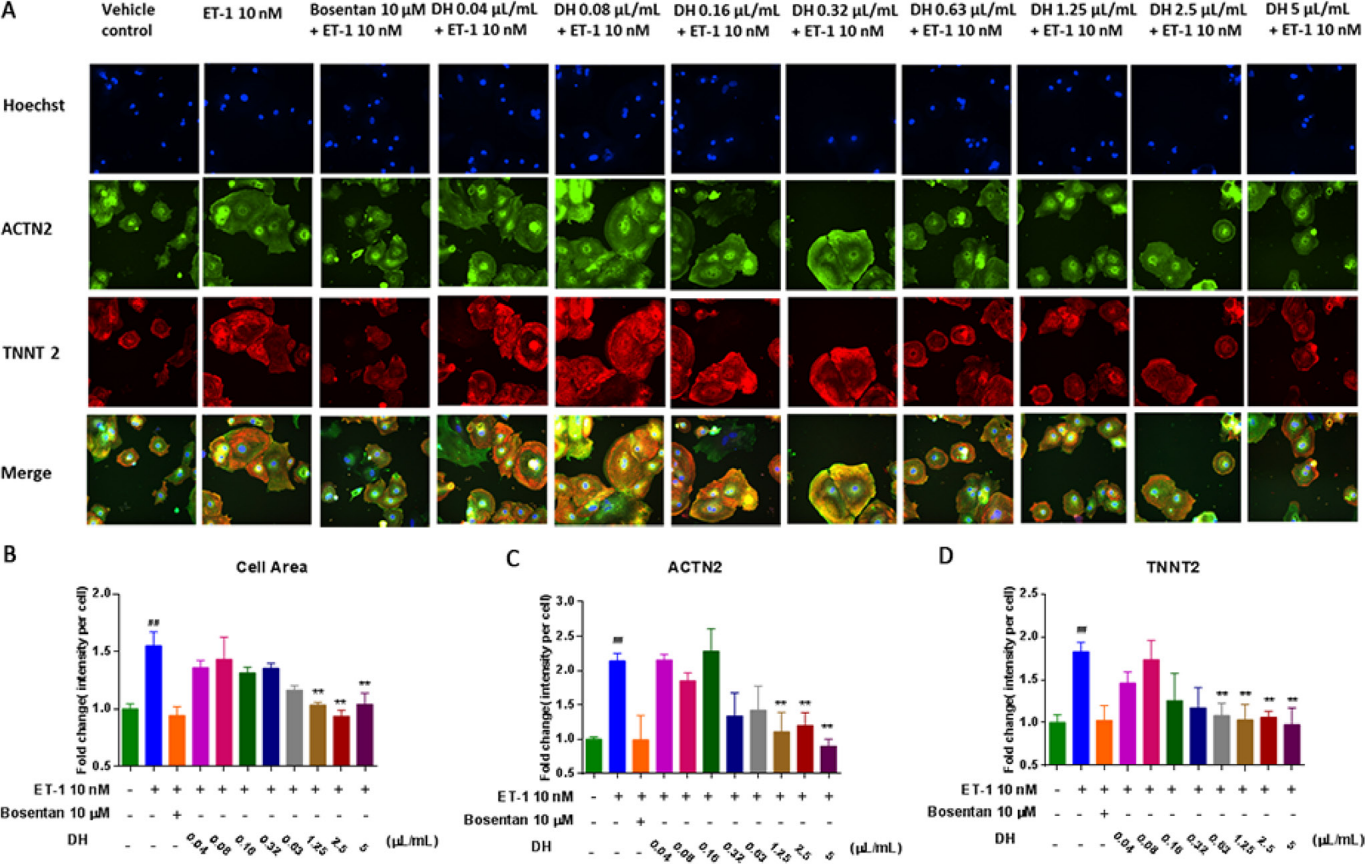
DH attenuates ET-1 induced cell area enlargement and elevation of ACTN2 and TNNT2 contents on hypertrophic hiPS-CMs (**A**) Representative images of the HCA analysis of hypertrophic hiPS-CMs treated by a various dose of DH for the evaluation of cell area, ACTN2 and TNNT2 contents. (**B**) Cell area of hypertrophic hiPS-CMs were alleviated by DH treatment at 1.25, 2.5 and 5.0 μL/mL. (**C**) ACTN2 content of hypertrophic hiPS-CMs were suppressed by DH treatment at 1.25, 2.5 and 5.0 μL/mL. (**D**) TNNT2 content of hypertrophic hiPS-CMs were suppressed by DH treatment at 0.63, 1.25, 2.5 and 5.0 μL/mL. ^**^P < 0.05: versus ET-1 group; ^##^*P* < 0.05: versus vehicle group.

In order to study the effect of DH on CH, cell area, actinin alpha 2 (ACTN2) and cardiac muscle troponin T (TNNT2) content in hiPS-CMs were evaluated. The representative images showed changes in these indexes under different DH concentrations (Figure 2A). Cell area of hypertrophic hiPS-CMs induced by ET-1 was significant increased compared with that vehicle group (*P* < 0.01). The increase in cell area was alleviated by DH at 1.25, 2.5, 5.0 μL/mL compared with that of model group (*P* < 0.05), (Figure 2B). ACTN2 is an actin-binding protein with multiple roles, which localized to the Z-disc and analogous dense bodies in cardiomyocytes [10]. TNNT2 is the tropomyosin-binding subunit of the troponin complex, which is located on the thin filament of striated muscles and regulates muscle contraction in response to alterations in intracellular calcium ion concentration [11]. The abnormal increase of ACTN2 and TNNT2 protein levels indicated CH and other myocardial injuries [12]. In the present study, the expression of these two biomarkers in model group were remarkably increased compared with vehicle group (*P* < 0.05). DH at 1.25, 2.5, 5.0 μL/mL reduced the increase of ACTN2 content induced by ET-1 in hiPS-CMs (*P* < 0.05) (Figure 2C). Moreover, TNNT2 content increase was inhibited by DH at 0.63, 1.25, 2.5, 5.0 μL/mL compared with that of model group (*P* < 0.05) (Figure 2D).

**Figure 3:**
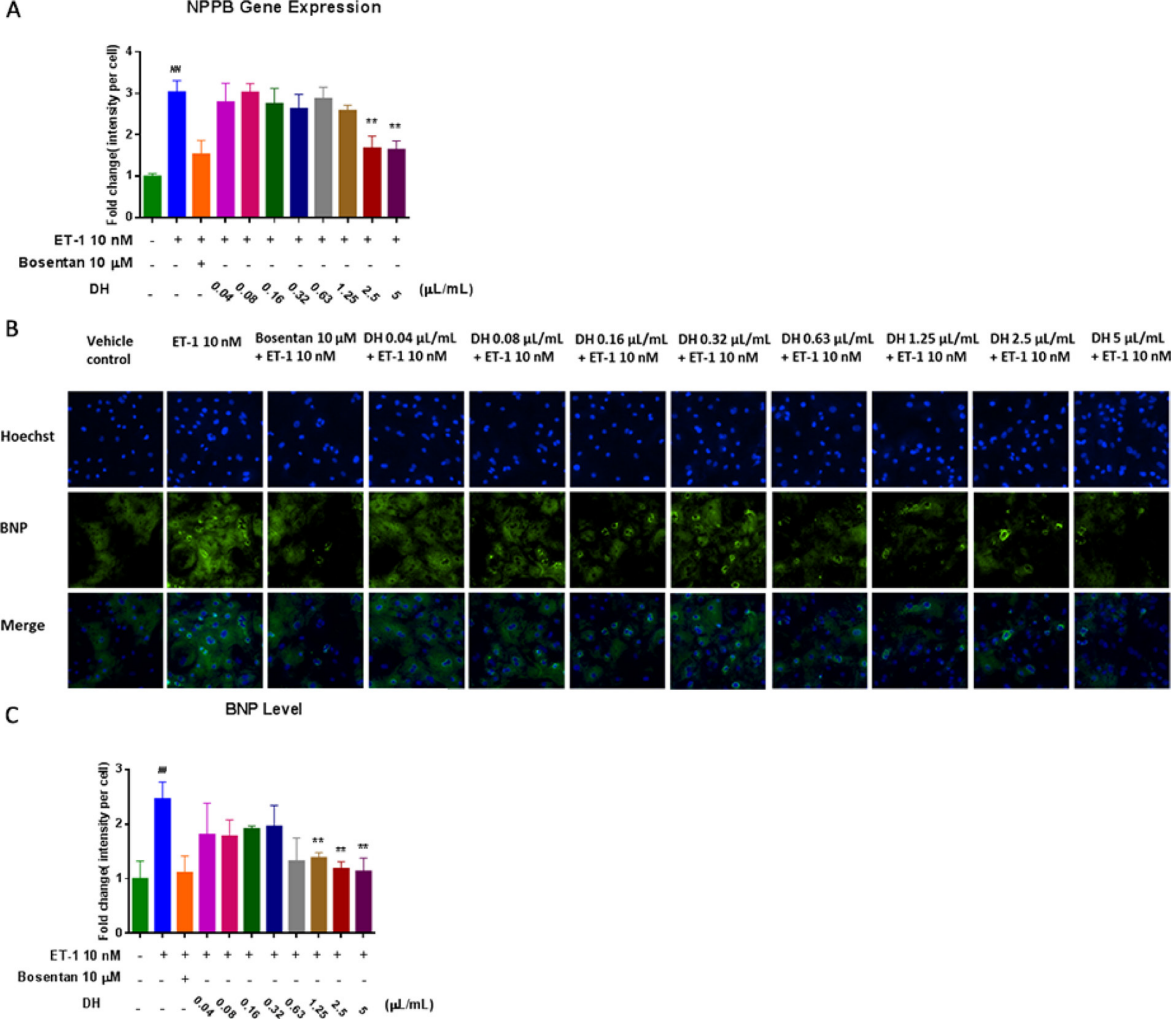
DH attenuated elevation of NPPB gene expression and BNP protein levels in CH (**A**) NPPB gene expression of hypertrophic hiPS-CMs were suppressed by DH treatment at 2.5 and 5.0 μL/mL. (**B**) Representative images of the HCA analysis of hiPS-CMs treated by DH for the evaluation of BNP content. (**C**) BNP content of hypertrophic hiPS-CMs were suppressed by DH treatment at 1.25, 2.5 and 5.0 μL/mL. ^**^P < 0.05: versus ET-1 group; ^##^*P* < 0.05: versus vehicle group.

Natriuretic peptide precursor B (NPPB) gene is a member of the natriuretic peptide family and encodes brain natriuretic peptide (BNP) protein, which functions as a cardiac hormone. The protein plays a key role in cardiovascular homeostasis. A high BNP protein concentration in the bloodstream is indicative of HF [13]. To investigate the cardio-protective effect of DH, NPPB expression in hiPS-CMs was evaluated by RT-PCR. NPPB expression on hypertrophic hiPS-CMs was inhibited by DH at 2.5 and 5.0 μL/mL compared with that of ET-1 group (*P* < 0.01) (Figure 3A). BNP measurement has become a mainstay for HF diagnosis and monitoring worldwide [14]. Thus, HCA was adopted for the evaluation of BNP content (Figure 3B). BNP content increase in hypertrophic hiPS-CMs was inhibited by DH at 1.25, 2.5, 5.0 μL/mL compared with that of ET-1 group (*P* < 0.01) (Figure 3C).

### DH showed antagonism effects on HEK293/Gα15/ET_B_ and HEK293/Gα15/AT_1_ cell lines

**Figure 4:**
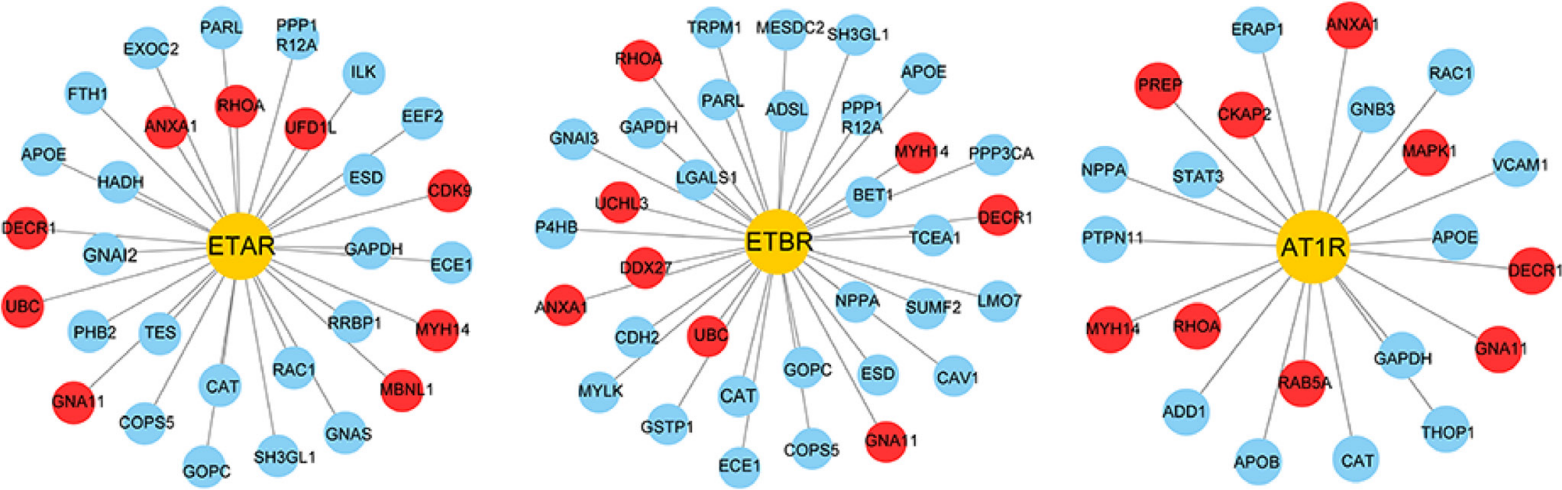
ET-1 related receptors and their interacting proteins Yellow nodes represent the receptors, blue nodes represent the interacting proteins. Red nodes represent differentially expressed proteins after DH treatment.

ET system is well established as a key player in the pathophysiology of various CVDs, such as pulmonary arterial hypertension and HF, which are accompanied by pathological CH [15, 16]. The deleterious effects of ET are mediated by both ET_A_ and ET_B_ receptors. There is an interaction between ET-1 and angiotensin II (Ang II). On the one hand, ET-1 increased angiotensin converting enzyme activity to promote Ang I transformation to Ang II. On the other hand, Ang II increased ET converting enzyme activity, and ET-1 production by elevating pre-endothelin mRNA transcription [17]. In our study, proteomics method was used to investigate protein expressions of hypertrophic cardiomyocytes after DH treatment. According to this data, there were 30, 34 and 22 proteins interacting with ET-1 related receptors (ET_A_R, ET_B_R and AT_1_R), which were also identified in proteome of hypertrophic cardiomyocytes after DH treatment. And some differentially expressed proteins interacted with ET-1 related receptors which indicated that DH treatment induced receptors related proteins differential expressing in ET-1 induced hypertrophic hiPS-CMs model (Figure 4).

**Figure 5:**
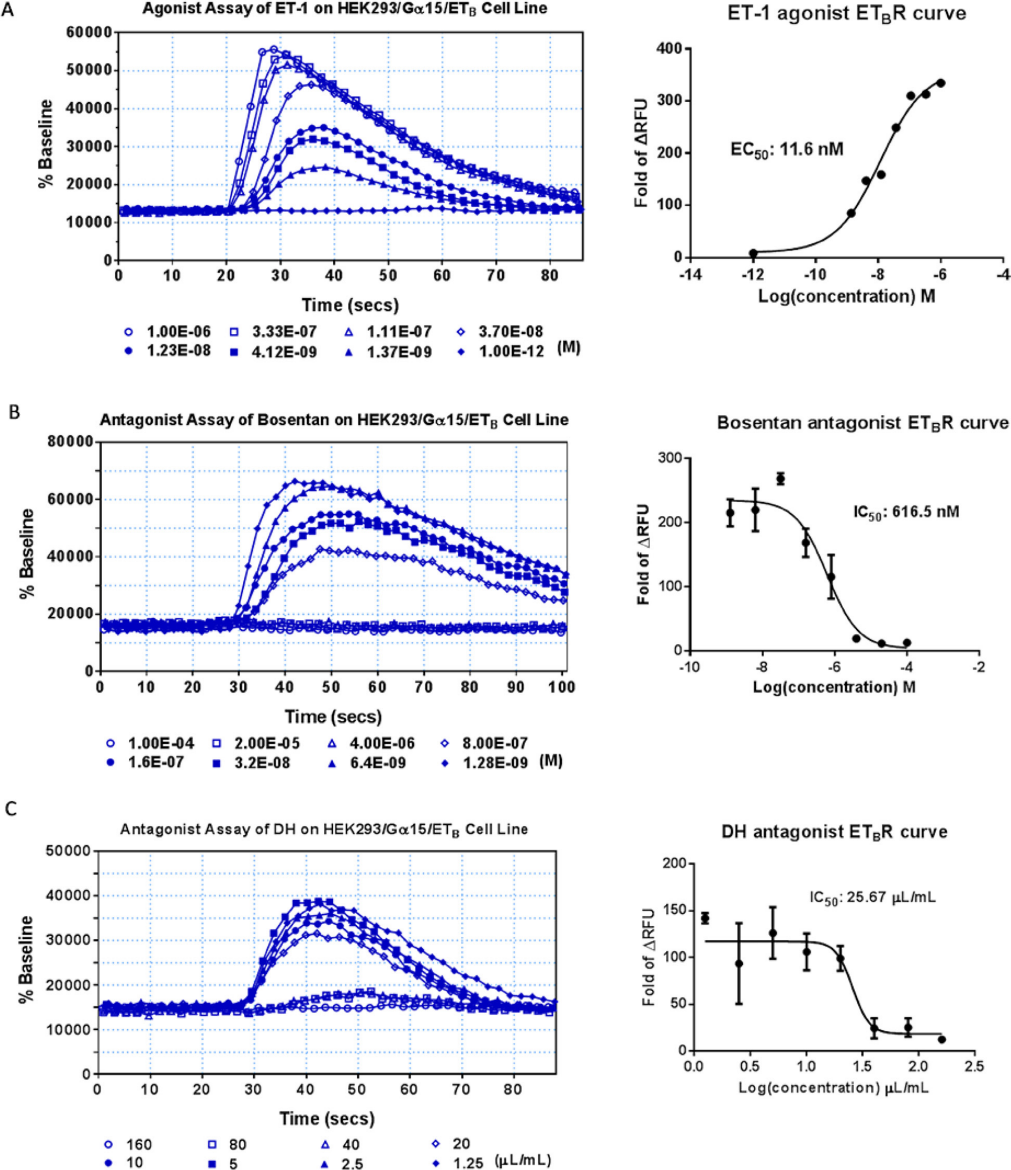
Agonist and antagonist assays of DH on HEK293/Gα15/ETB cell line (**A**) Agonist assay of the HEK293/Gα15/ETB cell line. Dose-response curve for ET-1, with EC50 value of 11.6 nM. (**B**) Antagonist assay of the HEK293/Gα15/ETB cell line. Dose-response curve for bosentan, with IC50 value of 616.5 nM. (**C**) Antagonist assay of DH on HEK293/Gα15/ETB cell line. Dose-response curve for DH, with IC50 value of 25.67 μL/mL.

The effects of DH on ET_A_R, ET_B_R and AT_1_R were detected by Flexstation II in the present study. The three receptors belong to the G-protein coupled receptors (GPCRs), assigned to Gq coupled receptors [18]. HEK293/Gα15/ET_A_ and HEK293/Gα15/ET_B_ cell lines were tested using known agonists and antagonists. DH was not an ET_A_R agonist or antagonist in the present study (Supplementary Figure 2), although it was identified as an ET_B_R antagonist. The EC50 value of ET-1, an ET_B_R reference agonist, was 11.6 nM (Figure 5A), and ET-1 signal could be inhibited by bosentan, an ET_B_R reference antagonist, in a dose-dependent manner with an IC50 value of 616.5 nM (Figure 5B), which was in accordance to previous reports [19]. DH showed antagonist effect on ET_B_R with an IC50 value of 25.67 μL/mL (Figure 5C). Moreover, DH was not an ET_B_R agonist (Supplementary Figure 3A).

**Figure 6:**
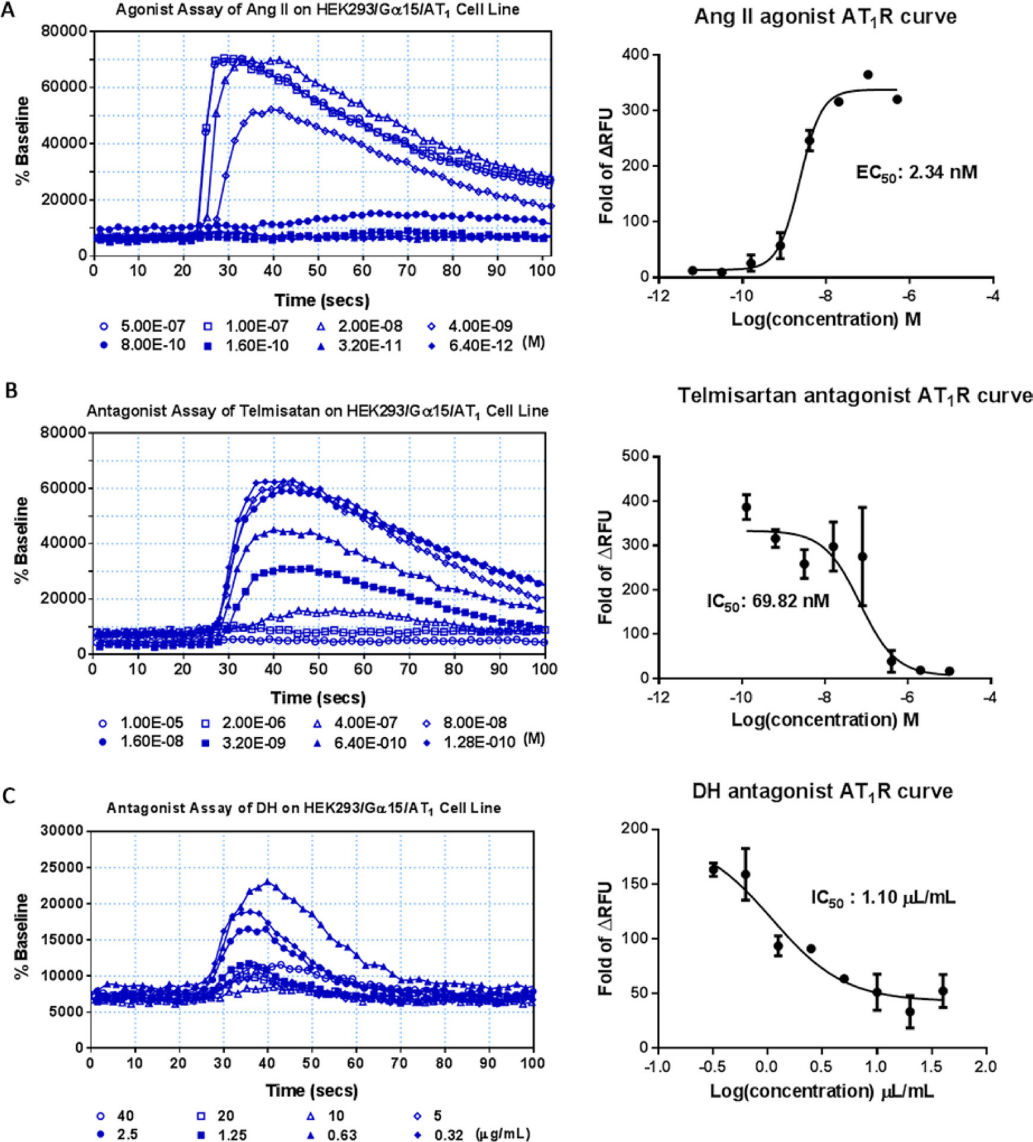
Agonist and antagonist assay of DH on HEK293/Gα15/AT1 cell line (**A**) Agonist assay of the HEK293/Gα15/AT1 cell line. Dose-response curve for angiotensin II, with EC50 value of 2.34 nM. (**B**) Antagonist assay of the HEK293/Gα15/AT1 cell line. Dose-response curve for telmisartan, with IC50 value of 69.82 nM. (**C**) Antagonist assay of DH on HEK293/Gα15/AT1 cell line. Dose-response curve for DH, with IC50 value of 1.10 μL/mL.

HEK293/Gα15/AT_1_ cell lines were validated with known agonists and antagonists. Consistent with previously published reports, EC50 value of angiotensin II, a AT_1_R reference agonist, was 2.34 nM (Figure 6A), and angiotensin II signal was inhibited by telmisartan, the AT_1_R antagonist, in a dose-dependent manner with an IC50 value of 69.82 nM (Figure 6B). DH was not an AT_1_R agonist (Supplementary Figure 3B), while it was an AT_1_R antagonist with an IC50 value of 1.10 μL/mL (Figure 6C).

### DH modulated the expression of proteins involved in cell cycle, RNA processing, cytoskeleton and mitochondrial translation

**Figure 7:**
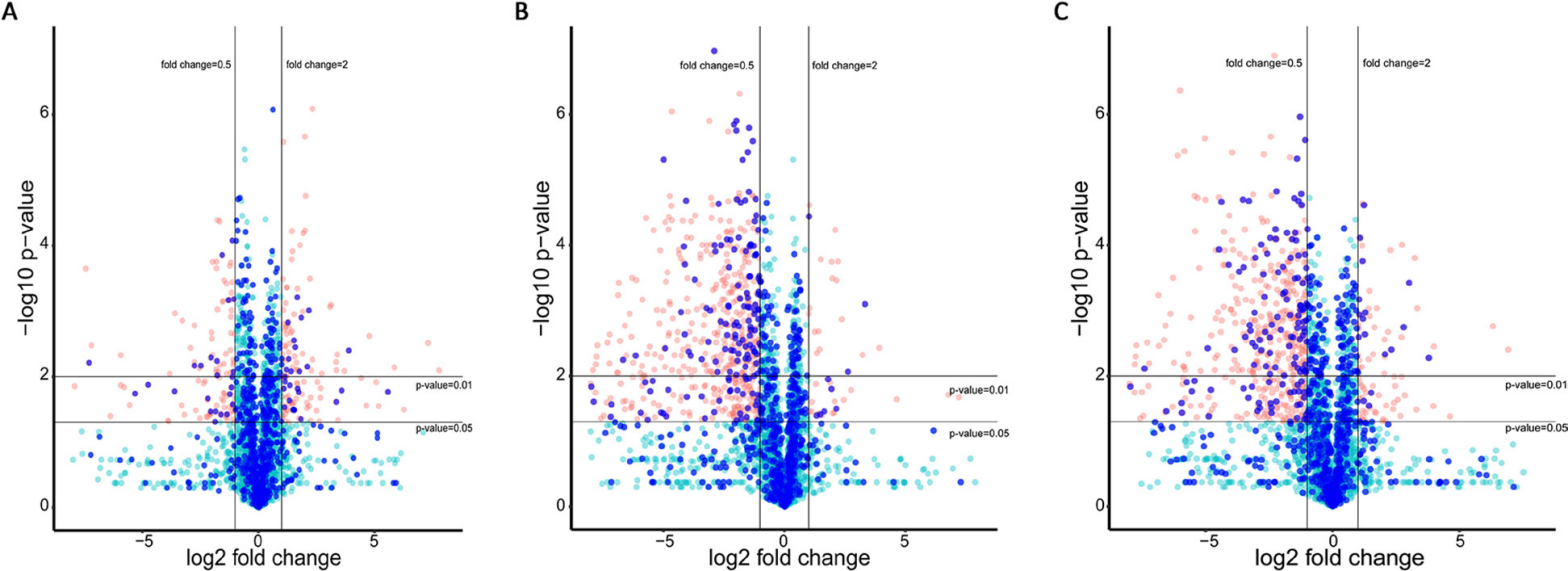
Protein expression of hypertrophic hiPS-CMs with different treatments Red dots represent differentially expressed proteins (fold change > 2 and *p*-value < 0.05), while green dots represent proteins without significant difference. CH related proteins and their interacting proteins are marked as blue. (**A**) Differential protein expression in ET1-induced CH model against control. (**B**) Differential protein expression after Bosentan treatment against ET1-induced CH model. (**C**) Differential protein expression after DH treatment against ET1-induced CH model.

To investigate the pathogenesis of CH, as well as DH mechanism of action, hypertrophic hiPS-CMs protein expression was analysed by proteomics method. A number of 2870 proteins were identified and quantified, and 716 of them were relevant to CH (CH related proteins and interacting partners). Differential expression analysis of proteins in hypertrophic hiPS-CMs showed significant change compared with that of vehicle control (Figure 7A). As a positive control, bosentan inhibited the abnormal expression increase induced by ET-1 (Figure 7B) and DH displayed an effect similar to bosentan (Figure 7C).

**Figure 8:**
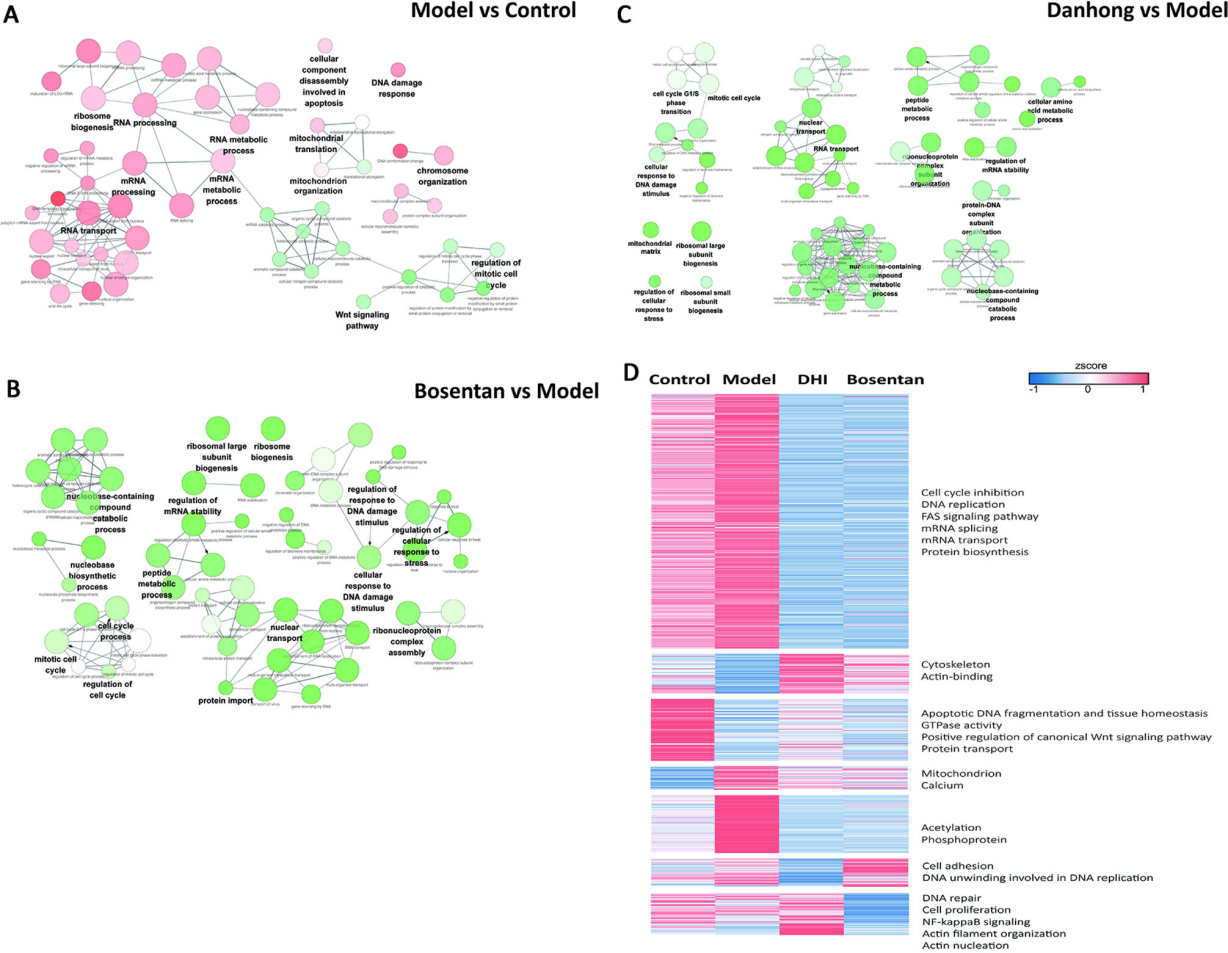
Biological process for differentially expressed proteins of hypertrophic hiPS-CMs Red nodes indicate that over 70% of the differentially expressed proteins associated with the term were up-regulated, and green nodes mean 70% of the differentially expressed proteins in this term were down-regulated. The color gradient of node represents the significance of over-representation, and size of node represents the number of protein in this term. (**A**) Over-represented GO terms (Biological Process at 4th level) for differentially expressed proteins identified from vehicle control and ET1-induced CH model. (**B**) Over-represented GO terms (Biological Process at 4th level) for differentially expressed proteins identified from model group and bosentan treatment by ClueGO. (**C**) Over-represented GO terms (Biological Process at 4th level) for differentially expressed proteins identified from model group and DH treatment by ClueGO. (**D**) Hierarchical clustering of the quantitative information from significantly differentially expressed proteins, expression level of proteins were normalized using z-score.

Gene Ontology (GO) enrichment analysis was used to evaluate the role of the proteins differentially expressed. On the one hand, the differentially expressed proteins having a role in RNA processing were up-regulated. On the other hand, the proteins differentially expressed involved in cell cycle and Wnt signaling pathway were down-regulated (Figure 8A). These results indicated that RNA processing was activated during CH pathological progress, while cell cycle related proteins were suppressed. Bosentan regulated the expression of proteins involved in several mechanisms, including DNA damage, cellular response to stress and cell cycle (Figure 8B). In the present research, DH played a similar role to bosentan, since it regulated the expression of proteins involved in the same mechanisms described above. Moreover, the differentially over-represented proteins involved in RNA transport and mitochondrial function were down-regulated by DH treatment compared with that of model group (Figure 8C). According to the different variation tendencies, proteins expression were grouped into seven categories with different treatments. For instance, the proteins related to mechanisms including cell cycle inhibition, RNA processes and intracellular calcium-flux, were up-regulated in model group, while down-regulated by DH treatment (Figure 8D).

**Figure 9:**
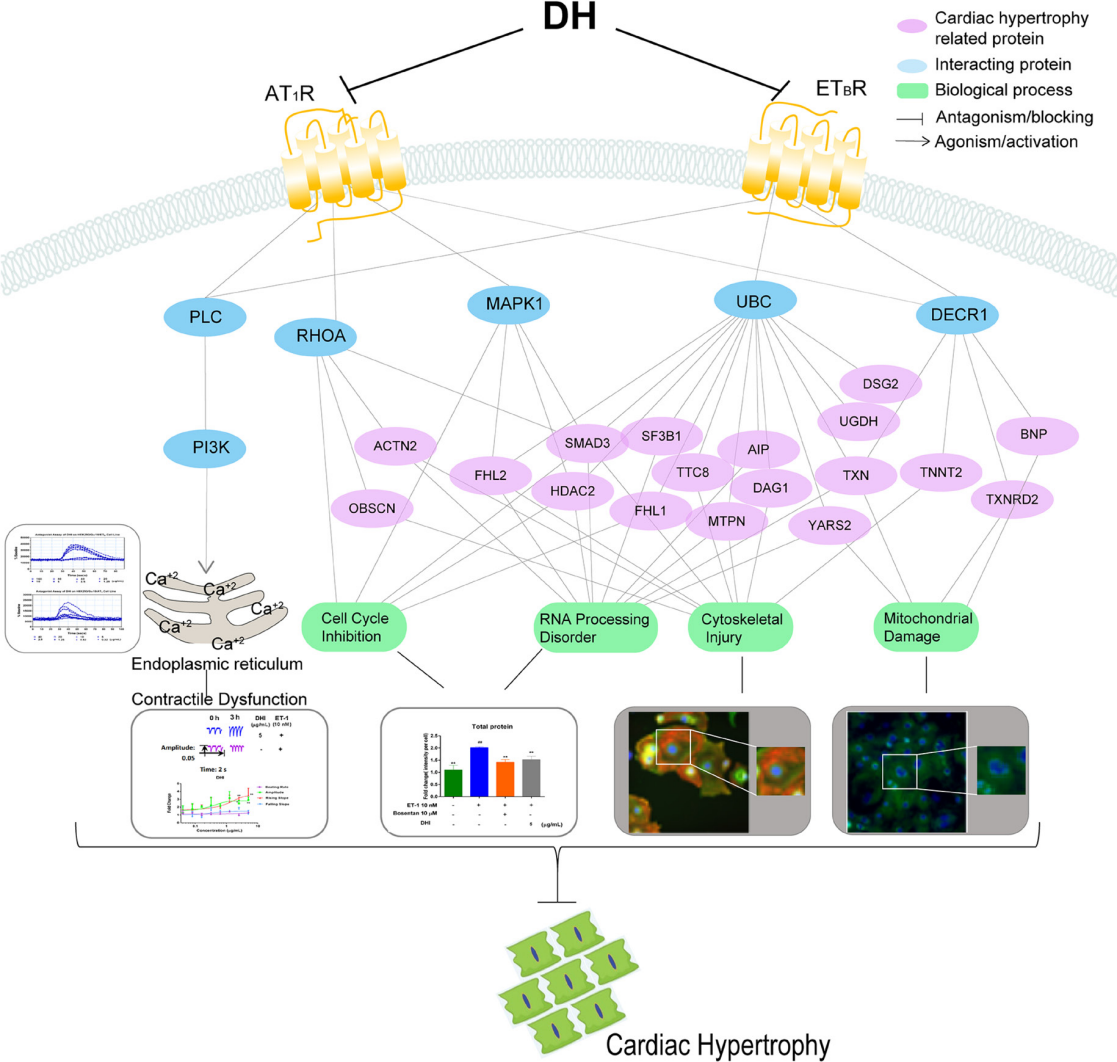
Schematic representation of DH-mediated protection from ET-1-induced CH on hiPS-CMs Signaling network after DH treatment. DH attenuated CH by blocking ET_B_R and AT_1_R, and then suppressing the downstream signaling pathways related to cell cycle, RNA processing, cytoskeleton and mitochondria. Blue nodes present the key proteins related to CH, and purple nodes presents proteins related to CH. Green node presents the biological processes.

To investigate how DH treatment influence CH, signal transduction network was constructed to present the interactions between ET-1 related receptor and CH proteins. Those interacting proteins were acquired from STRING database which outline some signal flows from DH treatment to biological process related with CH (Figure 9). ET-1 treatment induced up-regulation of some proteins and DH treatment made them down-regulated, and these protein with up-down manner were considered as the ones which were activated by ET-1 and antagonised by DH. Downstream signal pathways of ET_B_R and AT_1_R were consisted of abnormal expressed proteins related with CH, and other key proteins as Mitogen-activated protein kinase 1 (MAPK1), Ras homolog gene family, member A (RHOA), ubiquitin C (UBC) and 2,4-dienoyl-CoA reductase 1 (DECR1) (interaction score larger than 0.5). The further bioinformatics analysis showed that the CH related proteins participate in biological processes, such as cell cycle, cytoskeleton, mitochondria, RNA processing, calcium transportation and so on.

## DISCUSSION

DH has been included in the National Health Insurance Catalog of China for many years, and the indications are mainly referred to patients with severe acute ischemic cardiovascular and cerebrovascular diseases. On the basis of clinical results, some pharmacological studies on DH effect have been previously reported. For instance, DH exerts anti-hypertrophic effects on the heart by regulating p38 and NF-κb pathway [20]. Moreover, DH protective mechanism against H/R- and H_2_O_2_-induced injury is mediated by the inhibition of mPTP opening via mitigating Ca^2+^ overload and ROS generation [8]. In our previous research, pre-B-cell leukemia transcription factor 1 and cyclic AMP-dependent transcription factor 1, along with six other transcription factors, were supposed to be the putative target transcription factors for DH-mediated protection against cerebral ischemia [21]. To the best of our knowledge, millions of patients have been treated with DH in China. Although DH is a widely used clinical application, with extensive curative effects, the direct DH targets have not been discovered yet. In the present study, ET_B_R and AT_1_R were identified as DH direct targets.

ET_B_R is one of the G protein coupled receptor (GPCRs), which is located in many cell types including endothelial cells, cardiomyocytes, and protoplasmic astrocyte cells [22]. ET-1 is classically described as a peptide produced by the endothelium and cardiomyocytes, acting in a paracrine manner on ET receptors of the cells leading to constriction [23]. The structure analysis revealed that ET-1 induced conformational changes are propagated to the ET_B_R core and the cytoplasmic G-protein coupling interface, and probably induce conformational flexibility in TM6 [24]. Previously reports showed that ET_B_R induced vasorelaxation through nitric oxide (NO) and prostacyclin (PGI2) [25]. The overexpressed ET_B_R was observed in hypertension, ischemic heart disease, as well as cerebral ischemia [26]. These studies demonstrated that ET_B_R plays an important role in CVDs pathological processes. In the present study, DH was identified as an ET_B_R antagonist on hypertrophic hiPS-CMs. Thus, this outcome revealed that DH was exerting its protecting effect against CH and consequent diseases through ET_B_R. AT_1_R is one of the Gq-coupled receptor, located in several cell types including cardiomyocytes, neuronal cells and hepatocytes [27], which is mediating the majority of renin-angiotensin system responses like vasoconstriction, angiogenesis, and aldosterone synthesis [28]. AT_1_R crystal structure have also been analysed in previous studies [29]. AT_1_R up-regulation was observed in several diseases including heart failure, hypertension, diabetes and peripheral artery disease [30]. AT_1_R antagonists represent an effective therapy to attenuate hypertension and diabetic renal injury, and to improve heart failure. DH blocked AT_1_R during CH pathological process of CH in our present study, which might explain its effect on angina pectoris, ischemic cardiomyopathy, and dilated cardiomyopathy.

During CH pathological process, the disorder in calcium flow release results in systolic and diastolic dysfunction. In the present study, intracellular calcium flux were increased due to ET-1 stimulation. ET_B_R and AT_1_R, have been identified as the targets of many pharmacological antagonists in CVDs treatment [31] The activation of the receptors, which, in turn, activates phospholipase C (PLC) to catalyse the hydrolysis of phosphatidylinositol diphosphate to produce phosphoinositide 3-kinase (PI3K) and diglyceride. PI3K promotes the delivery of calcium in the muscle or endoplasmic reticulum to the cytoplasm, and the sustained calcium release induced the hypertrophic gene response. In our study, DH modulated the contractile dysfunction of hypertrophic cardiomyocytes by the antagonism on ET_B_R and AT_1_R.

As a GPCRs-activating molecule, ET-1 is linked to HF ominous progression [32]. This strongly suggests that once GPCRs are activated, cardiomyocytes undergo pathological hypertrophy through different signalling pathways. Several cardiomyocytes damages might be avoided or reduced through GPCRs antagonists. Our present study is an example supporting this aspect (Figure 9). The downstream proteins of pathways triggered by ET_B_R and AT_1_R, involving certain biological processes, were regulated. For instance, the differential protein expression in hypertrophic cardiomyocytes were mostly concerning cell cycle inhibition, RNA processing, cytoskeleton and mitochondrial translation.

In the present study, DH was identified as an ET_B_R and AT_1_R antagonist, and it regulated the downstream proteins of signalling pathways triggered by these receptors. The proteins involved in cell cycle were regulated by DH treatment. Previous study suggest that CH is due to cardiomyocytes hypertrophy and non-cardiomyocyte proliferation [33]. During pathological modifications, adult myocardial cells terminate differentiation, a lack of mitosis is observed and cells cannot enter the cell cycle [34]. Cell cycle changes play a vital role in the pathogenesis of CH. In the pathological process of CH, cardiomyocytes RNA processing was disturbed and resulted in further aggravated diseases. For instance, the proteins involved in the acceleration of RNA degradation were increased, while, the proteins reducing the degradation process were decreased. Furthermore, RNA transport was promoted in hypertrophic cardiomyocytes. RNA degradation, transport, splicing and metabolism were accelerated during CH pathological process. As a result, the total cardiomyocytes proteins were increased. In the present study, DH elevated the cardiomyocytes proteins involved in the cell cycle and RNA processing by its antagonism on ET_B_R and AT_1_R. As a result, DH suppressed the total protein increase of hypertrophic cardiomyocytes.

Moreover, DH attenuates cytoskeleton structure disorders and cell area increase of hypertrophic hiPS-CMs. As it known to all, cardiomyocytes have an intrinsic ability to sense and respond to mechanical load through a process known as mechanotransduction [35]. Pathological CH and HF are the results of maladaptive remodelling processes under prolonged and abnormal loading conditions. Human and mouse genetic studies have highlighted various cytoskeletal and sarcolemmal structures in cardiomyocytes as the likely candidates for load transducers. In our research, hiPS-CMs hypertrophy was accompanied by ACTN2 and TNNT2 content increase. Proteomics analysis revealed that more cytoskeletal proteins associated with CH were imbalanced, which might provide better insights into the mechanisms driving cytoskeleton-based cardiac diseases. DH suppressed morphological change, ACTN2 and TNNT2 content increase on hypertrophic hiPS-CMs by its antagonism on ET_B_R and AT_1_R, as well as regulated the proteins involved in the biological process of cytoskeleton structure.

CH is accompanied by changes in the metabolic substrates, energy consumption, and chronic energy deficiencies. The energy metabolism of the myocardium is related to the increase in the embryonic gene expression during CH and ventricular remodelling [36]. In our research, the proteins involved in mitochondrion translation and organization were abnormally expressed in hypertrophic cardiomyocytes. The evolution of CH to HF is accompanied by a decline in mitochondrial biological function and a decrease in oxidative phosphorylation [37]. Therefore, energy metabolism improvement can prevent or reverse pathological CH. Furthermore, emerging studies indicate that BNP is not only a well-known biomarker for HF, but also plays pivotal roles in metabolic control [38]. DH regulated the proteins involved in cardiomyocytes mitochondrial functions by its antagonism on ET_B_R and AT_1_R. Moreover, both NPPB gene and BNP protein expression increase on hypertrophic hiPS-CMs was down-regulated by DH treatment.

This study not only identified the direct target of DH, but also analyzed the association between the biological processes associated with CH. For the first time, our research provided a systematic analysis of the proteomics changes on hypertrophic hiPS-CMs.MAPK1, RHOA, UBC and DECR1were supposed to be the key proteins in ET_B_R and AT_1_R signal transduction pathways and their downstream effects. MAPK1 is involved in the pathological progress of ventricular dilation and cardiac dysfunction [39], while RHOA plays a role in CH and myocardial remodelling. It is worth mentioning that UBC and DECR1 are first considered to be associated with the pathogenesis of CH in our research. UBC is a polyubiquitin precursor. Conjugation of ubiquitin monomers or polymers can lead to various effects within a cell [40]. Ubiquitination has been associated with protein degradation, DNA repair, cell cycle regulation, and regulation of other cell signalling pathways (provided by RefSeq, Aug 2010). In the present research, UBC worked as a key protein in the pathological progress of CH, affecting with a series of proteins involved in cell cycle, RNA processing, cytoskeleton and mitochondria. DECR1 participates in the beta-oxidation and metabolism of unsaturated fatty enoyl-CoA esters [41]. In our study, this protein is involved in ET_B_R and AT_1_R signalling pathways on hypertrophic hiPS-CMs, interacting with the proteins involved in the processing of cytoskeleton structure and mitochondria function. Proteins interaction provided an explanation for the relationships between the pivotal processes.

**Figure 10:**
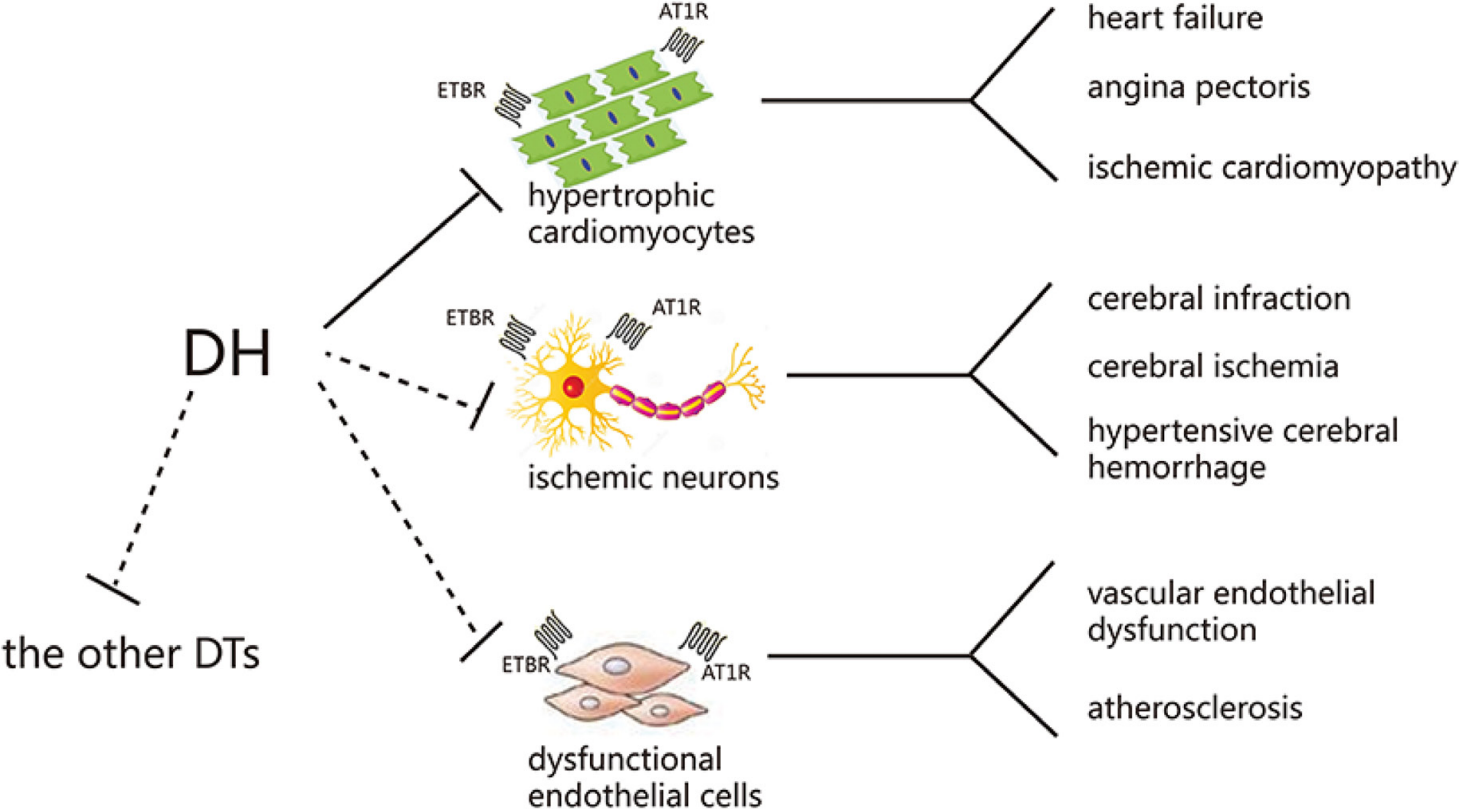
Possible mechanism of DH in treating cardiovascular and cerebrovascular diseases DTs: direct targets.

In conclusion, in the present study, ET_B_R and AT_1_R were identified as DH direct targets in hypertrophic hiPS-CMs. This research not only clarified DH protective mechanism, but also provided a strategy to explore CHMP direct targets. Furthermore, some work need be included in our research plan (Figure 10). On the one hand, the receptors expressed not only on cardiomyocytes, but also on the other cell lines, including neuronal cells, endothelial cells, and so on. There is a universal phenomenon, that is, a CHMP can treat more than one disease without any scientific proof on their mechanism of action. In clinical treatment, DH not only showed a therapeutic effect on CVDs, but also on cerebrovascular disease, reflecting the thought of “same treatment for different diseases” in CHMP. In our further research, other cell models will be employed. On the other hand, DH might effect on other receptors as a multiple components preparation. More other receptors validation should be added to the subsequent studies. Moreover, *in vivo* experiments will be considered in our future research to validate DH protective effect and detailed mechanism.

## MATERIALS AND METHODS

### DH and compounds preparation

In order to make sure the quality stability of DH and identify the main ingredients of DH, UltiMate 3000 UHPLC system equipped with a Q Exactive^™^ (Thermo Scientific^™^) was employed to analyse the multiple components in DH. DH was filtered through 0.45 μm filter before UHPLC analysis. The standard stock solutions of the standards were prepared by dissolving 5 mg of each compound in a 25 mL measuring flask with methanol respectively and analyzed directly. Separation was performed by UltiMate 3000 UHPLC system and screened with ESI-MS. The LC system was comprised of a waters T3 column (100 × 2.1 mm, 1.7μm C18). The mobile phase was composed of solvent A (0.1% formic acid-water) and solvent B (0.1% formic acid-acetonitrile) with a gradient elution (0–1 min, 5–5% B; 1–10 min, 5–95% B; 10–13 min, 95–95% B.). The flow rate of the mobile phase was 0.3 mL/min. The column temperature was maintained at 35°C, and the sample manager temperature was set at 4°C. The injection volume was 1 µL^-1^. Mass spectrometry was performed on a Q Exactive Mass Spectrometer (Thermo Scientific^™^, USA) using an ESI source. The instrument was operated in negative-ion modes. The scanning mass-to-charge (m/z) range was from 50 to 1500 with a scan rate of 1.00 spectra/sec. The spray voltage was 3700 KV. The capillary temperature: 320°C. The sheath gas was 30 psi. The aux gas was 10 arb.

DH was dissolved in DMEM at different concentrations (1:3200, 1:1600, 1:800, 1:400, 1:200, 1:100, 1:50, 1:25 by volume). DH dosage representation was 0.32, 0.63, 1.25, 2.5, 5, 10, 20, 40 μL/mL (crude drug weight/ culture medium volume). Quantities of crude drug were calculated to contain 750 g Salvia miltiorrhiza Bge. and 250 g tinctorius L. per litre [42].

ET-1 was purchased from R&D Systems (Minnesota, USA), and bosentan was purchased from Sigma-Aldrich Chemicals (St. Louis, Missouri, USA). All compounds were dissolved in DMSO before dilution in media for cell treatments. DMSO 0.1% was used as vehicle control.

### Cells and treatments

hiPS-CMs were obtained from CELLAPYBIO (Cat# CA2001106, Beijing, China), which are well validated cell line [43]. Briefly, each well of the 96-well plate was pre-coated with 50 μL of a 1:100 diluted matrigel solution (BD, New Jersey, USA) and maintained at 4°C overnight. Cells were seeded in the pre-coated 96-well plate at a density of 17,000 cells/well and routinely maintained at 37°C, 5% CO_2_. Cell culture medium was refreshed every 2 days. hiPS-CMs were pretreated with bosentan (10 μM) and DH. Then, CMs were exposed to ET-1 (10 nM) to induce hypertrophy. After 24 h incubation, cells were stained for BNP.

HEK293/Gα15 cells were provided by t HDBiosciences (Shanghai), and cultured in DMEM supplemented with 10% foetal bovine serum, 500 U/mL penicillin, and 500 μL/mL streptomycin in a humidified 5% CO_2_ incubator at 37°C. To generate cell lines stably expressing the human AT1 receptor gene, the coding region was subcloned into pcDNA3.1 (Invitrogen). The resulting construct was transfected into the HEK293/Gα15 cells using FuGene HD (Roche) following the manufacturer’s instructions. Selection of stably transfected cells was carried out in HEK293/Gα15 cells seeded in 6-cm dishes (Costar) by treatment with 800 μL/mL G418 (Sigma-Aldrich) dissolved in the culture medium. After 2 weeks incubation, single-cell active clones were isolated and AT1 receptor gene expression level was measured using fluorescent detection of calcium mobilization. The stably transformed clone that presented the highest AT1 receptor gene expression level was chosen for functional studies and named HEK293/Gα15/AT_1_ cell line. The cell line was maintained in culture medium supplemented with 400 μL/mL G418. Same protocol was performed to obtain HEK293/Gα15/ET_A_ cell line expressing the human ETA receptor gene, and HEK293/Gα15/ ET_B_ cell line expressing the human ETB receptor gene [44].

### Proteomics analysis (LC-MS/MS analysis)

Dried peptide samples were re-dissolved in solvent A (0.1% formic acid in water). Liquid chromatography - tandem mass spectrometry (LC-MS/MS) analysis was performed using Orbitrap Fusion mass spectrometer (Thermo Fisher Scientific) equipped with an online Easy-nLC 1000 nano-HPLC system (Thermo Fisher Scientific). The injected peptides were separated on a reverse-phase nano-HPLC C18 column (Pre-column: 3 μm, 120 Å, 2 cm × 100 μm ID; analytical column: 1.9 μm, 120 Å, 10 cm × 100 μm ID) at a flow rate of 400 nL/min with a 75-min gradient of 5 to 30% solvent B (0.1% formic acid in acetonitrile). For peptide ionization, 2000 V was applied and a 320°C capillary temperature was used. For the detection with Oribitrap Fusion mass spectrometry, a precursor scan was measured in the Orbitrap by scanning from m/z 300–1400 with a resolution of 120,000 (at m/z 200), target automatic gain control (AGC) value of 5e5 and a maximum injection time of 50 ms. Ions selected under top-speed mode were isolated in Quadrupole with an isolation width of 1.6 Da and fragmented by higher energy collision-induced dissociation (HCD) with normalized collision energy of 32%, then measured in the linear ion trap. Typical MS/MS scans mass spectrometric conditions were: targets AGC value of 5e3 and maximum fill time of 35 ms; dynamic exclusion was employed for 18 s. [45].

MS/MS spectra acquired on Oribitrap were searched against the human National Center for Biotechnology Information (NCBI) RefSeq protein databases (updated on 04-07-2013) using Maxquant (version 1.5.6.5). The parameter settings were the following: mass tolerance was set to 10 ppm for precursor and 0.5 Da for product ions; two missed cleavages were allowed; dynamic modifications set were acetyl(protein N-term), and oxidation(M); a false discovery rate (FDR) of 1% at both peptide and protein level was applied; enzyme specificity was set to “Trypsin”, and a minimum number of seven amino acids was required for peptide identification; proteins and protein isoforms that could not be discriminated by unique peptides were grouped into protein groups; match between runs was performed in three repeats [45].

### Evaluation of hiPS-CMs contraction by RTCA cardio system

xCELLigence RTCA cardio instrument was used to detect cardiomyocyte contraction. hiPS-CMs were plated onto 1 % matrigel –coated xCelligence E-Plate Cardio 96 at a density of 17,000 cells per well. The media was changed every other day after plating, using maintenance media. Media were exchanged 4 h before DH dilutions transferred to CMs on E-plate. Cells were preincubated with DH to be tested for 24 h before being exposed to ET-1 for 24 h. Signals were collected every 20 s. Data collection was controlled and analysed by the xCELLigence Cardio Software (Roche Diagnostics GmbH, Mannheim, Germany) which allows calculation of the CMs beating parameters such as cell index, beating rate, amplitude, beating patterns, rising slope and falling slope [46].

### Measurement of cell area, ACTN2, TNNT2, and BNP proteins by HCA

To assess changes in cell area, ACTN2, TNNT2, and BNP content in hiPS-CMs following exposure to the compounds mentioned below, ImageXpress XLS widefield HCA System was used. hiPS-CMs were pretreated with bosentan and DH. Then, CMs were exposed to ET-1 (10 nM). After 24 h incubation, ACTN2, TNNT2, and BNP proteins were identified on CMs. Briefly, CMs were fixed in 4% formaldehyde, permeabilized using 0.1% Triton-X100 (Sigma-Aldrich, Missouri, USA) in PBS, blocked in 5% BSA (Sigma-Aldrich, Missouri, USA) in PBS, and incubated overnight at 4 °C with Anti-ACTN2 antibody (Abcam, Cambridge, UK), Anti-TNNT2 antibody (Abcam, Cambridge, UK), Anti-BNP antibody (Abcam, Cambridge, UK). After washing, slides were incubated with anti-mouse IgM secondary antibody (Jackson Immuno Research, Pennsyivania, USA) for 1 h and then stained with Hoechst 33342 (Sigma-Aldrich, Missouri, USA) for nuclear detection. ACTN2, TNNT2, and BNP stained cells were visualized using ImageXpress XLS (Molecular Devices, California, USA). Images (40×) are representative of 4 independent studies [47].

### Measurement of NPPB gene expression by qRT-PCR

After exposure to the compounds, total RNA was isolated from hiPS-CMs, using TRIzol reagent (Invitrogen, USA) following the manufacturer’s instruction. Reverse transcription was performed using Revert Aid-First Strand cDNA Synthesis Kit (Fermentas, Canada). RT-PCR was performed using Taq DNA PCR Kit (Takara, Japan) in a 20 μL reaction volume on the qRT-PCR detection system (Funglyn, Canada). Primer sequences for the NPPB and β-actin were synthesized by Shanghai Sangon Biological Engineering Technology and Services Co. Ltd. NPPB-sense primer: 5′- CTGGC TGCTT TGGGA GGAA -3′; NPPB-antisense primer: 5′- AAAGG CGGCCACAGGGTTG-3′. β-actin gene was used as internal control. β-actin sense primer 5′-AATCTGGCACCACACCTTCTAC-3′; β-actin antisense primer 5′-ATAGCACAGCCTGGATAGCAAC-3′. All the primers were designed by PrimerBlast (GenBank). qRT-PCR reactions were as follows: initial denaturation at 95°C for 2 min, followed by 40 cycles of denaturation at 95°C for 15 sec, annealing at 56°C for 15 sec and extension at 72°C for 35 sec, with preservation carried out at 4°C. Reactions containing neither reverse transcriptase nor template were used as negative controls, and all the reactions were conducted in triplicate. The expression level was calculated by the 2^-ΔΔCT^ method, and subjected to statistical analysis.

### Calcium mobilization HTS assay

HEK293/Gα15/ET_A_, HEK293/Gα15/ET_B_, and HEK293/Gα15/AT_1_ cells were plated at a density of 40,000 cells/well in 96-well clear-bottom black plates (Costar) coated with matrigel (BD) and incubated at 37°C overnight. The following day, growth medium was removed and replaced with 100 μL assay buffer containing a final concentration of 4 μM calcium-sensitive dye Fluo-4-AM (Molecular Probes) and 2.5 mM probenecid (purity ≥ 98%, Sigma- Aldrich) in HBSS. Cells were incubated in a humidified atmosphere of 5% CO_2_ at 37 °C for 30 min. The intracellular Ca^2+^ flux was assayed using a fluorescent imaging plate reader (FlexStation II; Molecular Devices) to simultaneously monitor Fluo-4- AM fluorescence in all wells (λ excitation = 485 nm, λ emission= 525 nm). Cells were challenged with agonist ET-1 (100 nM), or angiotensin II (50 nM) (purity ≥ 98%, Sigma-Aldrich), and the fluorescence intensity was captured every 1.52 secs for 80 secs. For antagonist studies, the cells were preincubated with DH for 10 min prior to calcium-flux measurement; bosentan or telmisartan (purity ≥ 98%, Sigma- Aldrich) was used as a reference compound. Positive (1 μM angiotensin II) and negative (0.3% DMSO) controls were included in each 96-well plate for this assay. In addition, HEK293/Gα15/ET_A_, HEK293/Gα15/ET_B_, and HEK293/Gα15/AT_1_ cell line was tested for their response to the treatment with ET-1 or angiotensin II at every 5 passages for cell line stability test. The sample was identified as a primary hit when its inhibition rate was over 50%. For validation of the screening results, tested samples identified as hits in HTS were re-evaluated in triplicate [44].

### Compound specificity assay

HEK293 cell lines stably expressing human ET_B_R and AT_1_R genes provided by HDBiosciences (Shanghai), were separately plated at a density of 40,000 cells/well in 96-well clear-bottom black plates coated with matrigel and incubated at 37°C overnight. DH was tested at different concentrations (0.32, 0.63, 1.25, 2.5, 5, 10, 20, 40 μL/mL) using a calcium assay when cells were challenged with their selective agonists for antagonist identification. The parental HEK293 cells were used as the naive group, and cells were challenged with 10 μM ATP (purity ≥ 99%, Sigma-Aldrich) as an agonist (Supplementary Figure 4).

### Cytotoxicity assay

HEK293 cells were seeded at 10,000 cells/well into E-Plate and incubated at 37°C and 5% CO_2_. DH at different concentrations (0.32, 0.63, 1.25, 2.5, 5, 10, 20, 40 μL/mL) was added into each E-Plate well and incubated at 37°C and 5% CO_2_ for 24 h. Cell viability was detected by the addition of 100 μL CellTiter-Glo reagent (Promega, Madison, Wisconsin, USA) into each well containing cells in 100 μL medium and incubated for 10 minutes. Luminescence was read by Envision 2100 multilabel reader (PerkinElmer, Massachusetts, USA) [48]. Each experiment was performed with triplicates (Supplementary Figure 5A). Furthermore, the viability of hiPS-CMs were evaluated with the cell index by RTCA cardio system (Supplementary Figure 5B).

### Statistical analysis

Data were analysed using xCELLigence Cardio Software and further analysed with GraphPad Prism 6.0 (Graphpad Software, San Diego, California, USA). Data were expressed as mean ± SD, and statistical analysis was performed using ANOVA with LSD test. A value of 𝑃 < 0.05 was considered statistically significant.

Statistical analysis of differentially expressed proteins was performed using *T*-test to detect differentially expressed proteins based on three replications. Proteins with more than 2 fold change and 𝑃 < 0.05 were considered as differentially expressed proteins (marked as red in volcano plot) in a statistically significant manner. Volcano plot was used to summarize both fold-change and *t*-test criteria, which was a scatter-plot of the negative log10-transformed *p*-values from the test of proteins against the log2 fold change.

Enrichment analysis of differentially expressed proteins was based on the hyper-geometric cumulative distribution test, and the multiple testing correction was based on Benjamini-Hochberg correction method. Functional annotation of proteome data was performed using ClueGO [49] and visualized with Cytoscape v3.4.0 [50]. For the construction of “Receptors-interactors- hypertrophy related genes (HYPs)” signal transduction network, 277 myocardial HYPs were collected using the HPO term “left ventricular hypertrophy” [12] and “myocardial hypertrophy” related OMIM terms (Supplementary Table 2). Protein-protein interactions were retrieved from STRING database [51] and only relationships with confidence score larger than 0.5 (medium confidence) were taken into consideration. Network was constructed using co-expressed interactions between ET-1 antagonized receptors and their interactors, and co-expressed interactions between hypertrophy related proteins and their interactors. In addition, co-expressed interactions were defined based on the criteria - the trend of the relative expression for two interacting proteins are consistent in different conditions.

## SUPPLEMENTARY MATERIALS FIGURES AND TABLES





## Abbreviations

CH: cardiac hypertrophy
CVDs: cardiovascular diseases
DH: DanHong injection
CHMP: Chinese herbal medicine preparation
ET-1: endothelin-1
hiPS-CMs: human induced pluripotent stem cell-derived cardiomyocytes
RTCA: real time cellular analysis
HCA: high content analysis
HTS: high-throughput screening
BNP: brain natriuretic peptide
ACTN2: actinin alpha 2
TNNT2: cardiac muscle troponin T
ETBR: endothelin receptor type B
AT1R: angiotensin II receptor type 1
HF: heart failure
GO: gene ontology
GPCRs: G protein coupled receptor
PLC: phospholipase C
PI3K: phosphoinositide 3-kinase
MAPK1: mitogen-activated protein kinase 1
RHOA: ras homolog gene family, member A
UBC: ubiquitin C
DECR1: 2,4-dienoyl-CoA reductase 1

## References

[1] Li L, Xu J, He L, Peng L, Zhong Q, Chen L, Jiang Z (2016). The role of autophagy in cardiac hypertrophy. Acta Biochim Biophys Sin (Shanghai).

[2] Riaz S, Zeidan A, Mraiche F (2017). Myocardial proteases and cardiac remodeling. J Cell Physiol.

[3] Willenbrock R, Philipp S, Mitrovic V, Dietz R (2000). Neurohumoral blockade in CHF management. J Renin Angiotensin Aldosterone Syst.

[4] Zhiqiang L, Qingyuan J, Ma LJ, Yonghua JF, Chunyan H (2016). Impacts of Danhong Injection on the Concentrations of Fibrinogen and D-Dimeride in the Patients of Chronic Heart Failure. World Journal of Integrated Traditional and Western Medicine.

[5] Li M, Wang F, Huang Y, Du F, Zhong C, Olaleye OE, Jia W, Li Y, Xu F, Dong J, Li J, Lim JB, Zhao B (2015). Systemic exposure to and disposition of catechols derived from Salvia miltiorrhiza roots (Danshen) after intravenous dosing DanHong injection in human subjects, rats, and dogs. Drug Metab Dispos.

[6] Li X, Du F, Jia W, Olaleye OE, Xu F, Wang F, Li L (2017). Simultaneous determination of eight Danshen polyphenols in rat plasma and its application to a comparative pharmacokinetic study of DanHong injection and Danshen injection. J Sep Sci.

[7] Zhu J, Yi X, Huang P, Chen S, Wu Y (2017). Drug-protein binding of Danhong injection and the potential influence of drug combination with aspirin: Insight by ultrafiltration LC-MS and molecular modeling. J Pharm Biomed Anal.

[8] Duan ZZ, Li YH, Li YY, Fan GW, Chang YX, Yu B, Gao XM (2015). Danhong injection protects cardiomyocytes against hypoxia/reoxygenation- and H2O2-induced injury by inhibiting mitochondrial permeability transition pore opening. Journal of Ethnopharmacology.

[9] Xi B, Wang T, Li N, Ouyang W, Zhang W, Wu J, Xu X, Wang X, Abassi YA (2011). Functional cardiotoxicity profiling and screening using the xCELLigence RTCA Cardio System. J Lab Autom.

[10] Ribeiro Ede A, Pinotsis N, Ghisleni A, Salmazo A, Konarev PV, Kostan J, Sjoblom B, Schreiner C, Polyansky AA, Gkougkoulia EA, Holt MR, Aachmann FL, Zagrovic B (2014). The structure and regulation of human muscle alpha-actinin. Cell.

[11] Frey N, Luedde M, Katus HA (2011). Mechanisms of disease: hypertrophic cardiomyopathy. Nat Rev Cardiol.

[12] Kostin S, Hein S, Arnon E, Scholz D, Schaper J (2000). The cytoskeleton and related proteins in the human failing heart. Heart Fail Rev.

[13] Lippi G, Sanchis-Gomar F (2016). Monitoring B-type natriuretic peptide in patients undergoing therapy with neprilysin inhibitors. An emerging challenge?. Int J Cardiol.

[14] Torres-Courchoud I, Chen HH (2016). B-type natriuretic peptide and acute heart failure: Fluid homeostasis, biomarker and therapeutics. Rev Clin Esp.

[15] Tanaka A, Yuasa S, Mearini G, Egashira T, Seki T, Kodaira M, Kusumoto D, Kuroda Y, Okata S, Suzuki T, Inohara T, Arimura T, Makino S (2014). Endothelin-1 induces myofibrillar disarray and contractile vector variability in hypertrophic cardiomyopathy-induced pluripotent stem cell-derived cardiomyocytes. J Am Heart Assoc.

[16] Rehsia NS, Dhalla NS (2010). Potential of endothelin-1 and vasopressin antagonists for the treatment of congestive heart failure. Heart Fail Rev.

[17] Chang YK, Choi H, Jeong JY, Na KR, Lee KW, Choi DE (2016). Co-inhibition of Angiotensin II Receptor and Endothelin-1 Attenuates Renal Injury in Unilateral Ureteral Obstructed Mice. Kidney Blood Press Res.

[18] Salazar NC, Chen J, Rockman HA (2007). Cardiac GPCRs: GPCR signaling in healthy and failing hearts. Biochim Biophys Acta.

[19] Lehmann LH, Stanmore DA, Backs J (2014). The role of endothelin-1 in the sympathetic nervous system in the heart. Life Sci.

[20] Mao HP, Wang XY, Gao YH, Chang YX, Chen L, Niu ZC, Ai JQ, Gao XM (2016). Danhong injection attenuates isoproterenol-induced cardiac hypertrophy by regulating p38 and NF-kappab pathway. J Ethnopharmacol.

[21] Wei J, Zhang Y, Jia Q, Liu M, Li D, Zhang Y, Song L, Hu Y, Xian M, Yang H, Ding C, Huang L (2016). Systematic investigation of transcription factors critical in the protection against cerebral ischemia by Danhong injection. Sci Rep.

[22] Chen Y, Zhang H, Liu E, Xu CB, Zhang Y (2016). Homocysteine regulates endothelin type B receptors in vascular smooth muscle cells. Vascul Pharmacol.

[23] Schorlemmer A, Matter ML, Shohet RV (2008). Cardioprotective signaling by endothelin. Trends Cardiovasc Med.

[24] Shihoya W, Nishizawa T, Okuta A, Tani K, Dohmae N, Fujiyoshi Y, Nureki O, Doi T (2016). Activation mechanism of endothelin ETB receptor by endothelin-1. Nature.

[25] Rodriguez-Pascual F, Busnadiego O, Lagares D, Lamas S (2011). Role of endothelin in the cardiovascular system. Pharmacol Res.

[26] Oikonomidis DL, Baltogiannis GG, Kolettis TM (2010). Do endothelin receptor antagonists have an antiarrhythmic potential during acute myocardial infarction? Evidence from experimental studies. J Interv Card Electrophysiol.

[27] Durik M, Seva Pessoa B, Roks AJ (2012). The renin-angiotensin system, bone marrow and progenitor cells. Clin Sci (Lond).

[28] Ibarra C, Vicencio JM, Varas-Godoy M, Jaimovich E, Rothermel BA, Uhlen P, Hill JA, Lavandero S (2014). An integrated mechanism of cardiomyocyte nuclear Ca(2+) signaling. J Mol Cell Cardiol.

[29] Zhang H, Han GW, Batyuk A, Ishchenko A, White KL, Patel N, Sadybekov A, Zamlynny B, Rudd MT, Hollenstein K, Tolstikova A, White TA, Hunter MS (2017). Structural basis for selectivity and diversity in angiotensin II receptors. Nature.

[30] Kumar R, Thomas CM, Yong QC, Chen W, Baker KM (2012). The intracrine renin-angiotensin system. Clin Sci (Lond).

[31] Siryk-Bathgate A, Dabul S, Lymperopoulos A (2013). Current and future G protein-coupled receptor signaling targets for heart failure therapy. Drug Des Devel Ther.

[32] Bkaily G, Nader M, Avedanian L, Choufani S, Jacques D, D’Orleans-Juste P, Gobeil F, Chemtob S, Al-Khoury J (2006). G-protein-coupled receptors, channels, and Na+-H+ exchanger in nuclear membranes of heart, hepatic, vascular endothelial, and smooth muscle cells. Can J Physiol Pharmacol.

[33] Foglia MJ, Poss KD (2016). Building and re-building the heart by cardiomyocyte proliferation. Development.

[34] Szibor M, Poling J, Warnecke H, Kubin T, Braun T (2014). Remodeling and dedifferentiation of adult cardiomyocytes during disease and regeneration. Cell Mol Life Sci.

[35] Lyon RC, Zanella F, Omens JH, Sheikh F (2015). Mechanotransduction in cardiac hypertrophy and failure. Circ Res.

[36] Torrealba N, Aranguiz P, Alonso C, Rothermel BA, Lavandero S (2017). Mitochondria in Structural and Functional Cardiac Remodeling. Adv Exp Med Biol.

[37] Carvajal K, Moreno-Sanchez R (2003). Heart metabolic disturbances in cardiovascular diseases. Arch Med Res.

[38] He WT, Mori M, Yu XF, Kanda T (2016). Higher BNP levels within physiological range correlate with beneficial nonfasting lipid profiles in the elderly: a cross-sectional study. Lipids Health Dis.

[39] Chen QW, Edvinsson L, Xu CB (2009). Role of ERK/MAPK in endothelin receptor signaling in human aortic smooth muscle cells. BMC Cell Biol.

[40] Crinelli R, Bianchi M, Radici L, Carloni E, Giacomini E, Magnani M (2015). Molecular Dissection of the Human Ubiquitin C Promoter Reveals Heat Shock Element Architectures with Activating and Repressive Functions. PLoS One.

[41] Helander HM, Koivuranta KT, Horelli-Kuitunen N, Palvimo JJ, Palotie A, Hiltunen JK (1997). Molecular cloning and characterization of the human mitochondrial 2,4-dienoyl-CoA reductase gene (DECR). Genomics.

[42] He Y, Wan H, Du Y, Bie X, Zhao T, Fu W, Xing P (2012). Protective effect of Danhong injection on cerebral ischemia-reperfusion injury in rats. J Ethnopharmacol.

[43] Liang P, Lan F, Lee AS, Gong T, Sanchez-Freire V, Wang Y, Diecke S, Sallam K, Knowles JW, Wang PJ, Nguyen PK, Bers DM, Robbins RC (2013). Drug screening using a library of human induced pluripotent stem cell-derived cardiomyocytes reveals disease-specific patterns of cardiotoxicity. Circulation.

[44] Liu Q, Liu J, Guo H, Sun S, Wang S, Zhang Y, Li S, Qiao Y (2013). [6]-gingerol: a novel AT(1) antagonist for the treatment of cardiovascular disease. Planta Med.

[45] Cox J, Mann M (2008). MaxQuant enables high peptide identification rates, individualized p.p.b.-range mass accuracies and proteome-wide protein quantification. Nat Biotechnol.

[46] Yu Y, Sun S, Wang S, Zhang Q, Li M, Lan F, Li S, Liu C (2016). Liensinine- and Neferine-Induced Cardiotoxicity in Primary Neonatal Rat Cardiomyocytes and Human-Induced Pluripotent Stem Cell-Derived Cardiomyocytes. Int J Mol Sci.

[47] Zhang M, Wu H, Guo F, Yu Y, Wei J, Geng Y, Wang S, Li S, Yang H (2017). Identification of active components in Yixinshu Capsule with protective effects against myocardial dysfunction on human induced pluripotent stem cell-derived cardiomyocytes by an integrative approach. Mol Biosyst.

[48] Zhang MY, Yu YY, Wang SF, Zhang Q, Wu HW, Wei JY, Yang W, Li SY, Yang HJ (2017). Cardiotoxicity evaluation of nine alkaloids from Rhizoma Coptis. Human & Experimental Toxicology.

[49] Bindea G, Mlecnik B, Hackl H, Charoentong P, Tosolini M, Kirilovsky A, Fridman WH, Pages F, Trajanoski Z, Galon J (2009). ClueGO: a Cytoscape plug-in to decipher functionally grouped gene ontology and pathway annotation networks. Bioinformatics.

[50] Shannon P, Markiel A, Ozier O, Baliga NS, Wang JT, Ramage D, Amin N, Schwikowski B, Ideker T (2003). Cytoscape: a software environment for integrated models of biomolecular interaction networks. Genome Res.

[51] Szklarczyk D, Morris JH, Cook H, Kuhn M, Wyder S, Simonovic M, Santos A, Doncheva NT, Roth A, Bork P, Jensen LJ, von Mering C (2017). The STRING database in 2017: quality-controlled protein-protein association networks, made broadly accessible. Nucleic Acids Res.

