# Supplementary material for: DanHong injection targets endothelin receptor type B and angiotensin II receptor type 1 in protection against cardiac hypertrophy
Source: Oncotarget. 2017 Oct 13;8(61):103393–409. doi: 10.18632/oncotarget.21900 (PMC5732736; doi:10.18632/oncotarget.21900)
Supplement: Supplementary file 1 [file oncotarget-08-103393-s001.pdf]

DanHong injection targets endothelin receptor type B and angiotensin II receptor type 1 in protection against cardiac hypertrophy

SUPPLEMENTARY MATERIALS

Supplementary Table 1: Information for standard compounds in DHI

No.	Name	Retention Time (min)	Compound formula	Experimental m/z	Theoretical m/z	Error Δppm
1	tanshinol	4.51	C9H10O5	197.0445 [M-H]-	197.0455	−5.1
2	hydroxy safflor yellow A	4.87	C27H31O16	611.1617[M-H]-	611.1618	−0.2
3	protocatechuic aldehyde	5.12	C7H6O3	137.0230 [M-H]-	137.0244	−10.2
4	caffeic acid	5.32	C9H8O4	179.0339 [M-H]-	179.0350	−6.1
5	(2S, 3S)-1-Phenyl-2,3-butanediol-3-O-D-glucopyranoside	5.63	C17H26O9	373.1501 [M+COOH]−	373.1504	−0.8
6	salvianolic acid I	5.72	C27H22O12	537.1038 [M-H]-	537.1038	0.0
7	salvianolic acid D	5.88	C20H18O10	417.0821 [M-H]-	417.0827	−1.4
8	salvianolic acid E	5.98	C36H30O16	717.1459 [M-H]-	717.1461	−0.3
9	rosmarinic acid	6.16	C18H16O8	359.0769 [M-H]-	359.0772	−0.8
10	salvianolic acid B	6.25	C36H30O16	717.1455 [M-H]-	717.1461	−0.8
11	salvianolic acid A	6.48	C26H22O10	493.1135 [M-H]-	493.1140	−1.0

Supplementary Table 2: Hypertrophy related genes from HPO and OMIM. See Supplementary_Table_2

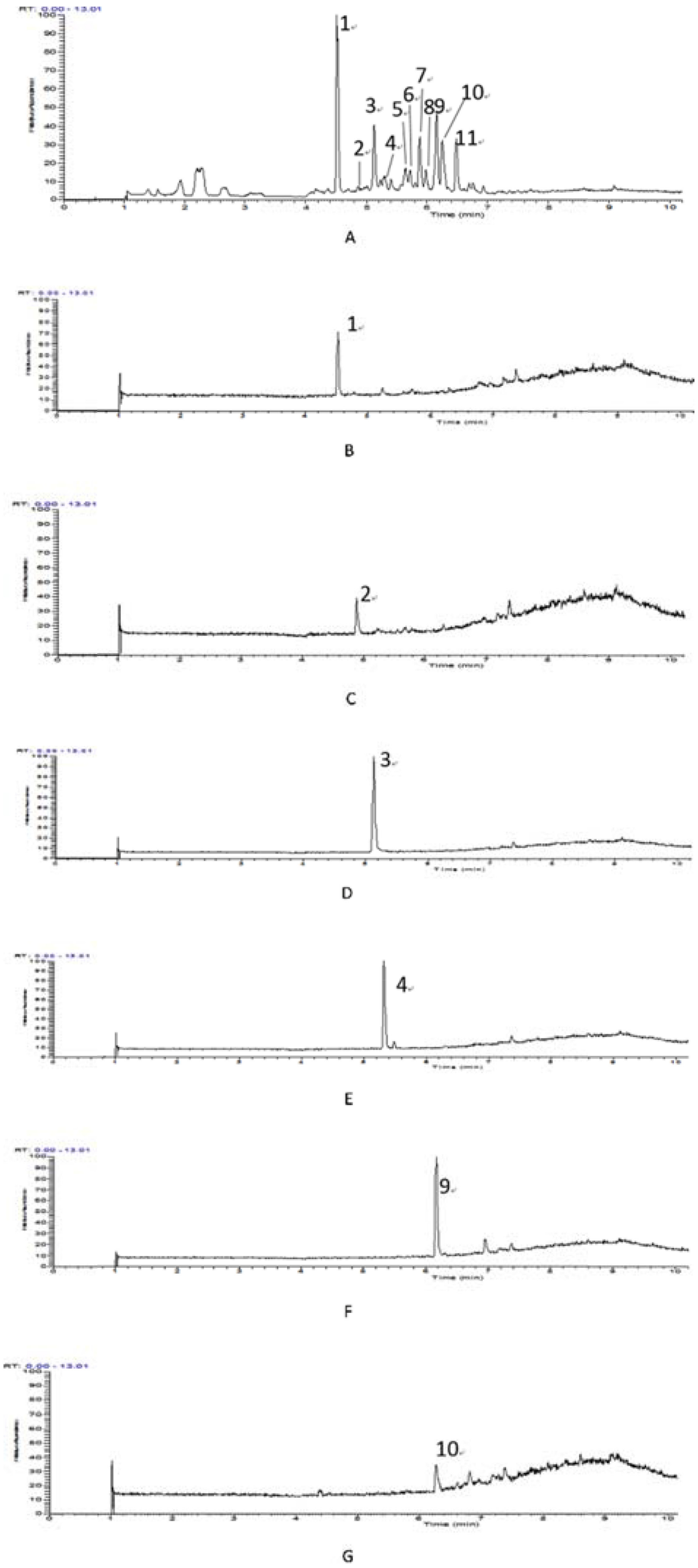

Supplementary Figure 1: The typical total ion chromatography (TIC) profles of DanHong injection (DH) and the standards. (**A**) TIC of DH; (**B–G**) TIC of the standards. 1 tanshinol; 2 hydroxy safflor yellow A; 3 protocatechuic aldehyde; 4 caffeic acid; 5 (2S, 3S)-1-Phenyl-2,3-butanediol-3-O-D-glucopyranoside; 6 Salvianolic acid I; 7 Salvianolic acid D; 8 Salvianolic acid E; 9 rosmarinic acid (10); 10 salvianolic acid B; 11 salvianolic acid A.

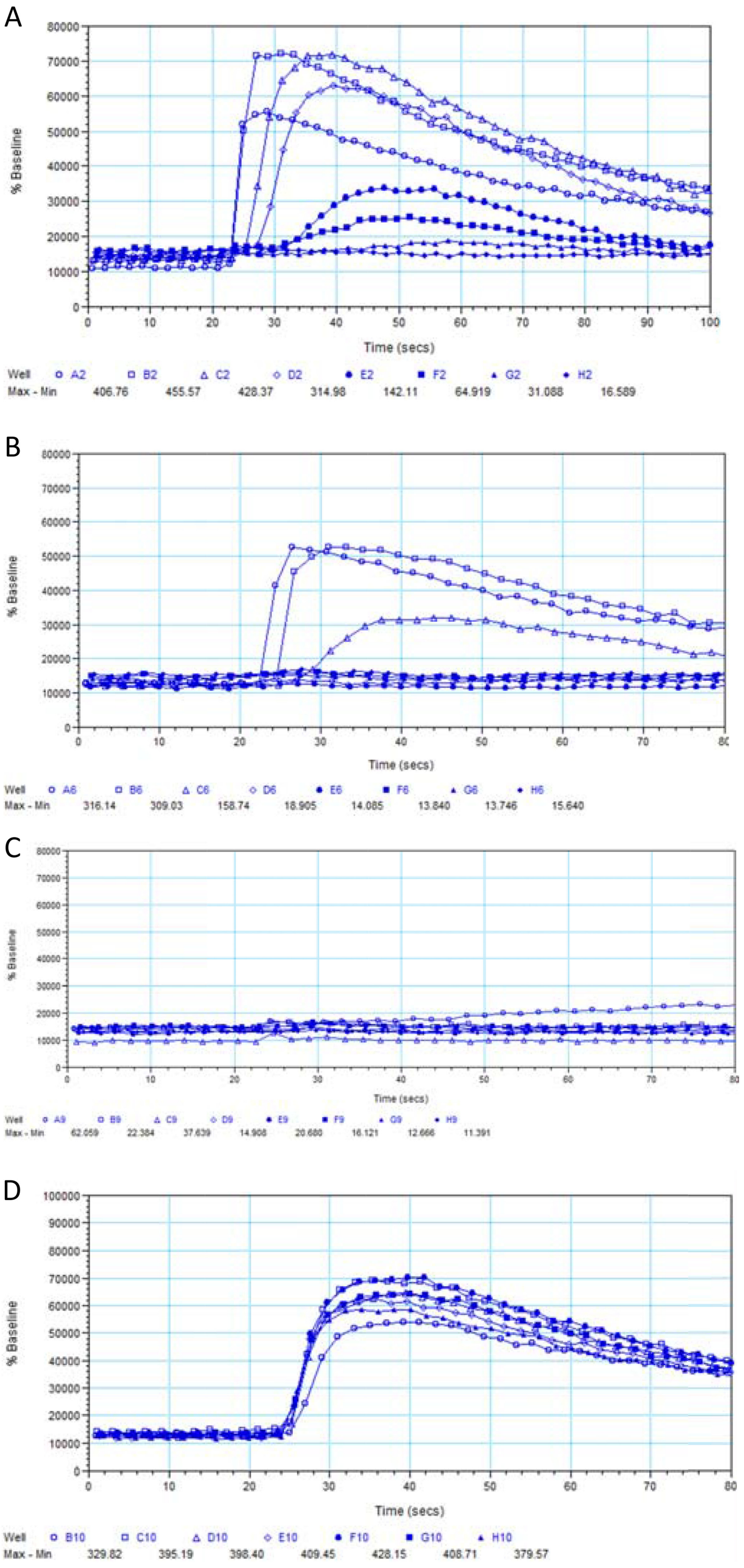

Supplementary Figure 2: Agonist and antagonist assays of DH on HEK293/Gα15/ETA cell line. (**A**) Agonist assay of the HEK293/Gα15/ETA cell line. Dose-response curve for ET-1. (**B**) Antagonist assay of the HEK293/Gα15/ETA cell line. Dose-response curve for bosentan. (**C**) Agonist assay of DH on HEK293/Gα15/ETA cell line. DH showed no agonism on ETAR. (**D**) Antagonist assay of DH on HEK293/Gα15/ETA cell line. DH showed no antagonism on ETAR.

A

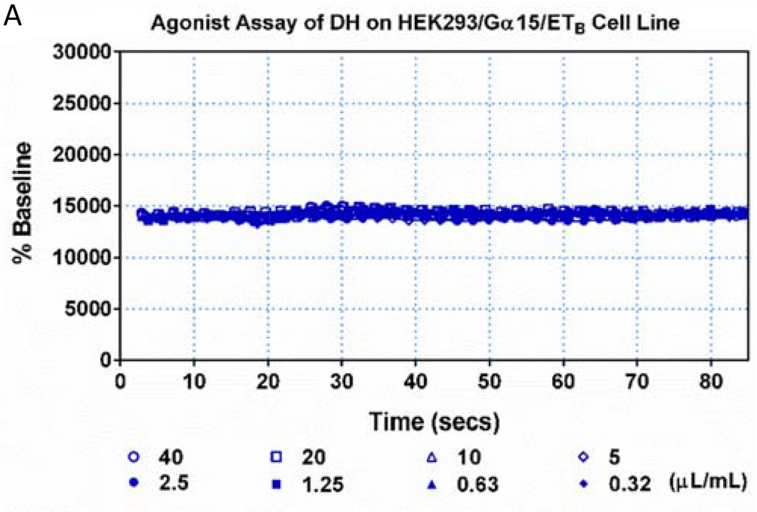

B

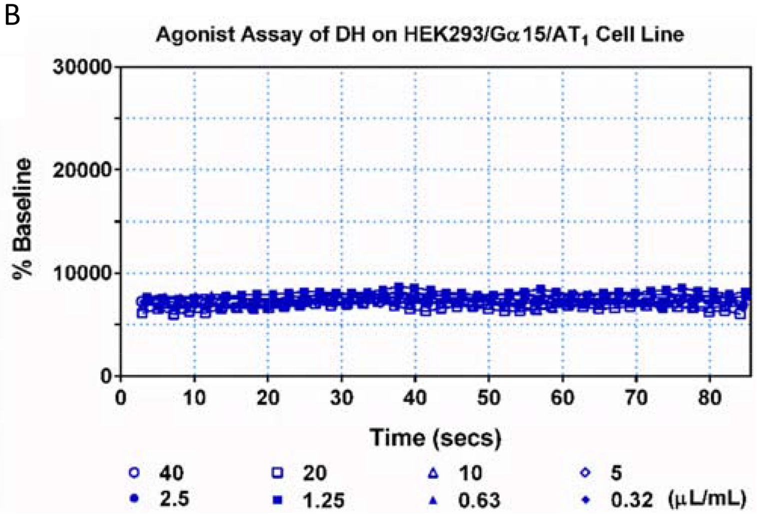

Supplementary Figure 3: Agonist assay of DH on HEK293/Gα15/ETB and HEK293/Gα15/AT1 cell lines. (**A**) Agonist assay of the HEK293/Gα15/ETB cell line. DH showed no antagonism on ET_BR. (**B**) Agonist assay of DH on HEK293/Gα15/AT1 cell line. DH showed no agonism on AT_1R.

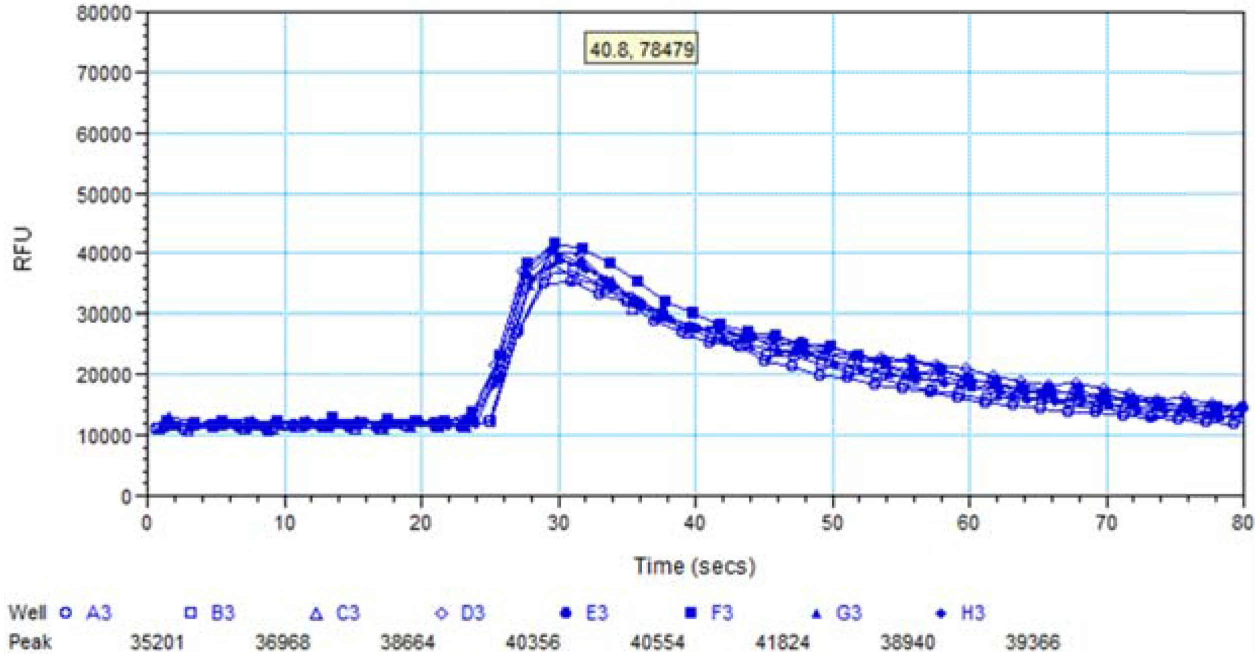

Supplementary Figure 4: Specificity assay of DH on HEK293 cell line.

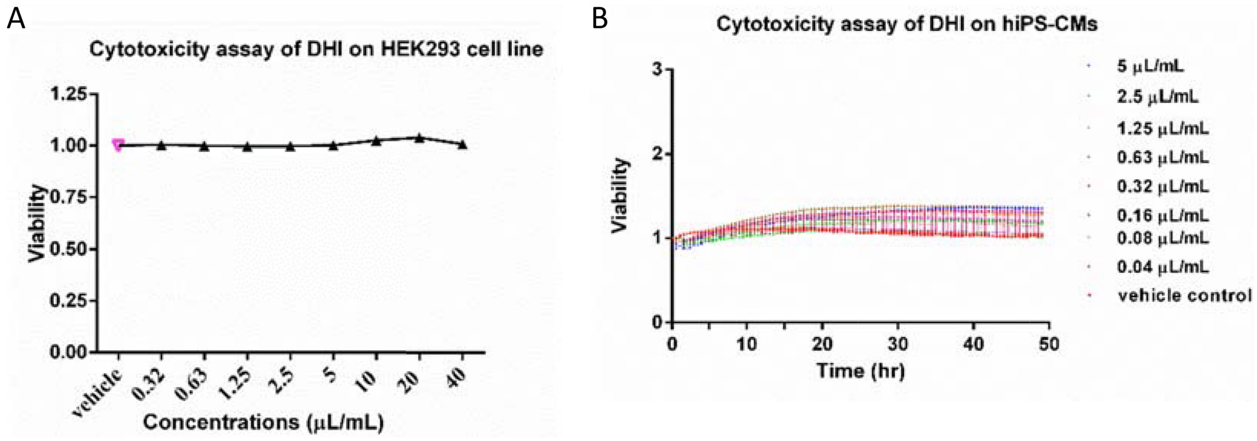

Supplementary Figure 5: Cell viability of hiPS-CMs incubated with DH. (**A**) ATP depletion of HEK293 cell line treated by DH. (**B**) Cell index of hiPS-CMs incubated with DH.
